# D6PK plasma membrane polarity requires a repeated CXX(X)P motif and PDK1-dependent phosphorylation

**DOI:** 10.1038/s41477-023-01615-6

**Published:** 2024-01-26

**Authors:** Alina Graf, Alkistis Eleftheria Lanassa Bassukas, Yao Xiao, Inês C. R. Barbosa, Julia Mergner, Peter Grill, Bernhard Michalke, Bernhard Kuster, Claus Schwechheimer

**Affiliations:** 1 Plant Systems Biology, School of Life Sciences, Technical University of Munich, Freising, Germany; 2 Proteomics and Bioanalytics, School of Life Sciences, Technical University of Munich, Freising, Germany; 3 Bavarian Center for Biomolecular Mass Spectrometry at Klinikum rechts der Isar, Center for Translational Cancer Research, Munich, Germany; 4 Helmholtz Zentrum München, German Research Center for Environmental Health, Analytical BioGeoChemistry, Neuherberg, Germany; 5 Present Address: Guangdong Provincial Key Laboratory of Plant Adaptation and Molecular Design, School of Life Sciences, Guangzhou University, Guangzhou, China

**Keywords:** Plant polarity, Auxin

## Abstract

D6 PROTEIN KINASE (D6PK) is a polarly localized plasma-membrane-associated kinase from *Arabidopsis thaliana* that activates polarly distributed PIN-FORMED auxin transporters. D6PK moves rapidly to and from the plasma membrane, independent of its PIN-FORMED targets. The middle D6PK domain, an insertion between kinase subdomains VII and VIII, is required and sufficient for association and polarity of the D6PK plasma membrane. How D6PK polarity is established and maintained remains to be shown. Here we show that cysteines from repeated middle domain CXX(X)P motifs are S-acylated and required for D6PK membrane association. While D6PK S-acylation is not detectably regulated during intracellular transport, phosphorylation of adjacent serine residues, in part in dependence on the upstream 3-PHOSPHOINOSITIDE-DEPENDENT PROTEIN KINASE, promotes D6PK transport, controls D6PK residence time at the plasma membrane and prevents its lateral diffusion. We thus identify new mechanisms for the regulation of D6PK plasma membrane interaction and polarity.

## Abstract (web summary)

PIN-FORMED auxin transporters are activated by D6PK, the localization of which depends on cysteine S-acylation. Phosphorylation by PDKs regulates D6PK transport and residence time, and prevents lateral diffusion, revealing novel mechanisms for membrane interaction and polarity.

## Main

The phytohormone auxin regulates essentially every aspect of plant growth and development. Auxin is transported, within the plant, from cell to cell by different auxin transporters^1^. The polar distribution of so-called ‘canonical’ PIN-FORMED (PIN) auxin efflux carriers can give directionality to auxin transport, and PIN polarity or activity regulation can explain auxin-controlled responses during development^1–4^.

‘Canonical’ PINs are activated by phosphorylation through serine/threonine kinases of the AGC1 and AGC3 families, kinases related to animal cyclic adenosine monophosphate- (cAMP-), cyclic guanosine monophosphate- (cGMP-) and Ca^2+^-dependent kinases^5–7^. The *Arabidopsis thaliana* AGC1 kinases D6 PROTEIN KINASE (D6PK), the three D6PK-LIKE1 (D6PKL1) to D6PK-LIKE3 (D6PKL3) kinases and PROTEIN KINASE ASSOCIATED WITH BREVIS RADIX (PAX)^5,8–12^ have been implicated in several auxin transport-dependent developmental and tropic growth processes, including phototropic hypocotyl bending^8,9,11,12^. Like their PIN phosphorylation substrates, D6PK, D6PKLs and PAX are polarly distributed at the plasma membranes of many cells^10,11,13,14^. D6PK, D6PKLs and PAX traffic very rapidly, and independent of PINs, to and from the plasma membrane by vesicular trafficking, with the help of the Brefeldin A (BFA) sensitive guanine exchange factor (GEF) GNOM^10,11,13^. How kinase polarity and trafficking are established and regulated remain to be demonstrated.

PDK1 (3-PHOSPHOINOSITIDE-DEPENDENT PROTEIN KINASE1) and the paralogous PDK2 activate D6PK and PAX through phosphorylation at a conserved activation loop serine-methionine-serine (SMS) motif^15,16^. *pdk1 pdk2* phenotypes can be explained by reduced D6PK and PAX activity, and an activation loop phosphorylation-mimicking serine-methionine-aspartate (SMD) mutation in PAX is sufficient to rescue the protophloem defect of *pdk1 pdk2*^16^.

AGC1 to AGC4 kinases are recognizable by their middle domain, an approximately 30–80 amino acid insertion between kinase subdomains VII and VIII^7,13,17^. The D6PK middle domain is required and sufficient for its polar plasma membrane targeting and contains a polybasic lysine/arginine (K/R)-rich motif for electrostatic interactions with phosphatidylinositol 4-phosphates^7,13,18^. Ionic strength is, however, not sufficient to release D6PK from membranes; thus, additional mechanisms must exist that anchor the kinase in the plasma membrane and the membranes of transport vesicles, and there must be mechanisms regulating D6PK trafficking between these membranes and controlling D6PK polar distribution^10,13^.

In this Article, we find that cysteines of CXX(X)P motifs, repeated in the middle domain, are S-acylated and required for D6PK plasma membrane association. Furthermore, we find that PDK-dependent serine phosphorylation promotes D6PK transport, controls D6PK residence time at the plasma membrane and prevents its lateral diffusion. We thus identify new mechanisms regulating D6PK plasma membrane interaction and polarity.

## Results

### Repeated CXX(X)P motifs are conserved in AGC1 kinases

**Fig. 1:**
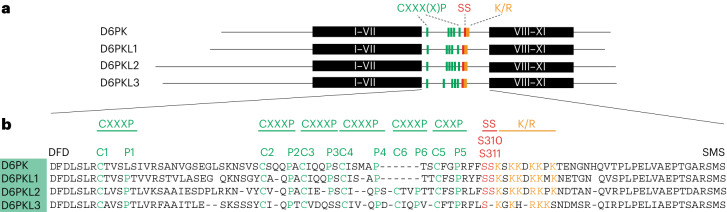
D6PK and D6PKLs share repeated CXX(X)P motifs and conserved serine residues. **a**,**b**, Schematic representation of full-length D6PK and D6PKL kinases from *A. thaliana* (**a**) and muscle alignment of their middle domain sequences (**b**) with the repeated CXX(X)P motifs, as well as adjacent serine residues, D6PK S310 and S311, and the polybasic motif (K/R) highlighted. In **a**, the conserved protein kinase subdomains I–VII and VIII–XI are shown as black boxes, and the remaining protein is shown as a line. In **b**, three residues of the flanking kinase subdomains VII (DFD) and VIII (SMS sequence of the activation loop) are included in the alignment to better delineate the middle domain.

The middle domain is required and sufficient for D6PK plasma membrane polarity^13^. Besides the K/R-rich motif, the D6PK middle domain contains five repeated CXX(X)P motifs, each composed of a highly conserved cysteine (C) and a proline (P) separated by two or three non-conserved amino acids (Fig. 1)^7^. While D6PKL1 has also five CXX(X)P motifs, designated here as C1XXXP1 to C5XXP5, D6PKL2 and D6PKL3 contain even six such motifs (Fig. 1). In D6PK, and in disagreement with the motif signature, C1XXXP1 lacks proline P1, but C1 can be unequivocally identified as C1 based when aligned with the other D6PKLs (Fig. 1b).

**Extended Data Fig. 1:**
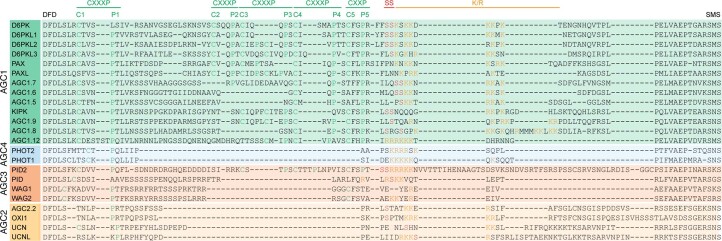
CXX(X)P motif repeats are a highly specific feature of AGC1 kinases. Muscle alignment of the middle domain sequences of *Arabidopsis thaliana* AGC1 – AGC4 kinases with cysteine (C) and proline (P) residues of CXX(X)P motifs, as well as adjacent serine (S) residues and polybasic motifs (K/R) highlighted. Three residues of the flanking kinase subdomains VII (DFD) and VIII (SMS sequence of the activation loop) are included in the alignment to better delineate the middle domain.

CXX(X)P motifs are present in all *Arabidopsis* AGC1 kinases, but their number varies between two and six CXX(X)P motifs in AGC1.5 and D6PKL2 or D6PKL3, respectively (Extended Data Fig. 1)^7^. Among the AGC2 to AGC4 kinases, many but not all family members have a single CXX(X)P motif corresponding to C1XXXP1, which could indicate that C1XXXP1 is evolutionarily the most ancestral motif (Extended Data Fig. 1). Among the AGC3 kinases, only PID-LIKE2 (PID2) has four CXX(X)P repeats and, based on this criterion, is more similar to AGC1 than to AGC3 kinases^7^. The numbers and also relative positions of the CXX(X)P motifs are highly conserved among paralogous AGC1 kinases from different plant species, suggesting functional importance (Supplementary Data Fig. 1).

### Cysteine but not proline residues are critical for D6PK plasma membrane association

**Fig. 2:**
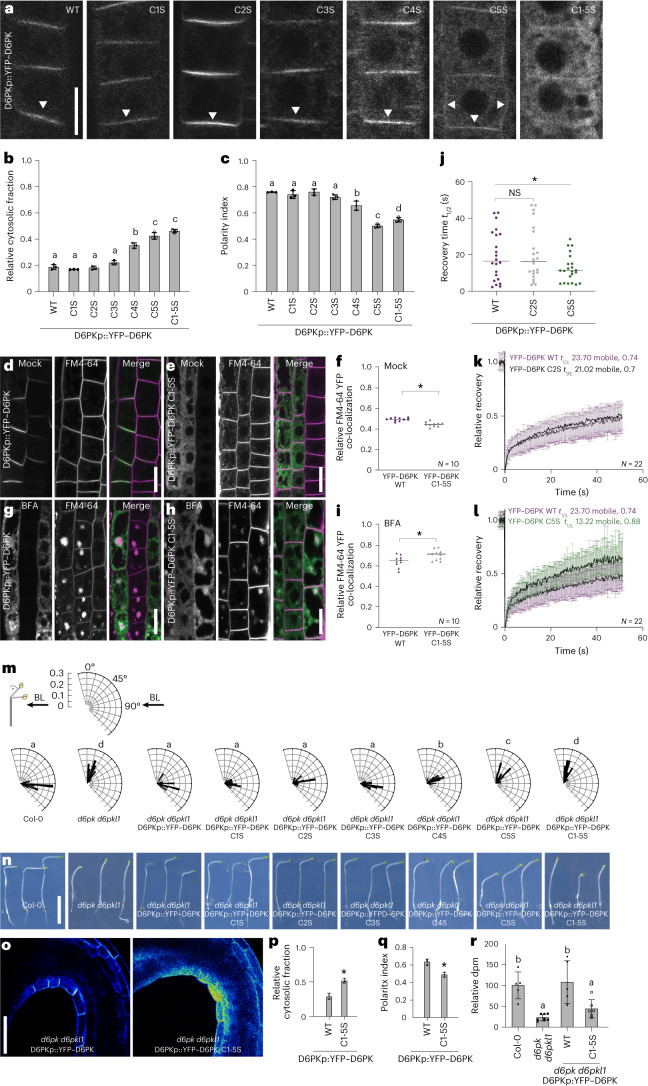
CXX(X)P motif cysteine mutations impair D6PK cellular distribution and D6PK-mediated phototropic hypocotyl bending. **a**, Representative confocal images of root epidermal cells from 6-day-old seedlings expressing YFP–D6PK from D6PKp::YFP–D6PK or variants with individual cysteines (C1S to C5S) or all five cysteines mutated to serines (C1-5S) in *d6pk d6pkl1*. Scale bar, 20 µm. **b**,**c**, The means and standard deviations of the relative cytoplasmic fractions (**b**) and the polarity indices (**c**) as determined from images as shown in **a**. The three data points represent the means of five measurements from one root, representing a biological replicate. **d**,**e**,**g**,**h**, Representative confocal images of root epidermal cells from 5-day-old seedlings after mock treatment and FM4-64 staining of D6PKp::YFP–D6PK (**d**) and of D6PKp::YFP–D6PK C1-5S (**e**) or after BFA treatment (10 µM for 15 min; 1 h 50 µM CHX pre-treatment) and FM4-64 staining of D6PKp::YFP–D6PK (**g**) and of D6PKp::YFP–D6PK C1-5S (**h**). Scale bars, 20 µm. **f**,**i**, Individual co-localization measures from ten cells depicted by the data points and the median of Pearson’s correlations of YFP and FM4-64 signals after mock (**f**) and BFA (**i**) treatments at the plasma membrane and in the cytosol. **j**–**l**, Results from FRAP experiments performed on epidermal cells of 5-day-old seedlings expressing the YFP-D6PK C2S (k) and YFP-D6PK C5S (l) transgenes in *d6pk d6pkl1*. The means and standard deviations of recovery times *t*_1/2_ at the basal plasma membrane (**j**) (*N* = 22 individual cells from three independent experiments) and the normalized recovery curves (**k**,**l**) (*N* = individual cells from three independent experiments as specified in the figure panels). **m**, Rose diagrams with the frequency distribution in 5° intervals of hypocotyl bending angles of >60 three-day-old dark-grown seedlings after unilateral blue light (BL) exposure for 4 h (arrow). **n**, Photographs of three representative seedlings from the experiment validated in **m**. Scale bar, 0.5 cm. **o**, Representative confocal images of the apical hook region of transgenic seedlings expressing the transgenes as specified in the *d6pk d6pkl1* background. Scale bar, 50 µm. **p**,**q**, The means and standard deviations of the relative cytoplasmic fractions (**p**) and the polarity indices (**q**) as determined from images as shown in **o**. The three data points represent the means of five measurements from one root, representing a biological replicate. **r**, The mean and standard deviation of basipetal auxin transport as measured in 4-day-old dark-grown seedlings with the specified genotypes. In **b**, **c** and **m**, the statistically significant difference between groups was determined by a two-tailed Student’s *t*-test (**f**,**i**,**j**,**p**,**q**) or a one-way ANOVA (**b**,**c**,**m**,**r**); means were compared using a Tukey’s test (**b**, *F*(6, 63) = 197.8, *P* < 0.0001; **c**, *F*(6, 84) = 104.6, *P* < 0.0001; **m**, *F*(8, 550) = 211.8, *P* < 0.0001; **r**, *F*(3, 20) = 12.27, *P* < 0.0001). Different letters indicate a statistically significant difference. Student’s *t*-test: NS, not significant; *P < 0.05.

**Extended Data Fig. 2:**
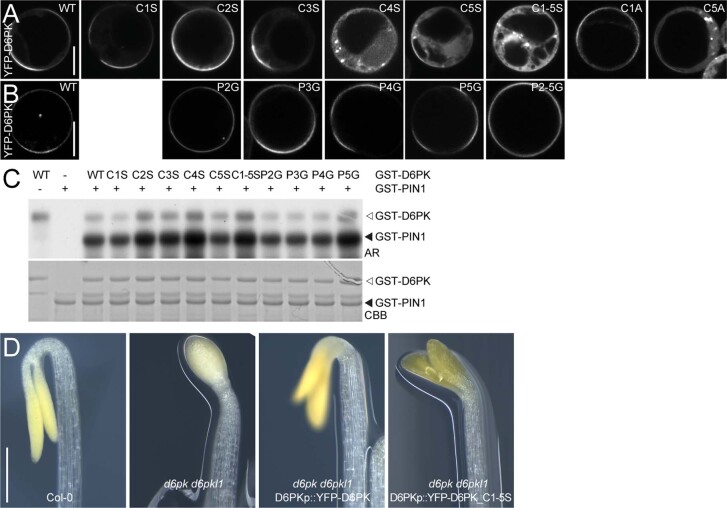
CXX(X)P motif cysteines but not prolines are important for plasma membrane association and auxin transport but do not impact D6PK kinase activity. **A** and **B**. Representative confocal images of transiently transformed protoplasts overexpressing wild type YFP-D6PK from 35Sp::YFP-D6PK or its mutant variants with individual or all five cysteines (C) mutated to serines (S) or alanine (A) (panel A) or with individual or all four prolines (P) mutated to glycines (G) (panel B). Scale bars = 10 µm. **C**. Result from *in vitro* kinase assays with recombinant wild type and mutant GST-D6PK as specified using a cytoplasmic loop fragment of PIN1 in GST-PIN1 as trans-phosphorylation substrate. AR, autoradiography; CBB, Coomassie Brilliant Blue-stained gel as loading control. This experiment was repeated three times with similar results. **D**. Representative images of the apical hook region of three-days old dark-grown seedlings with the specified genotypes. Scale bar = 0.5 mm.

**Extended Data Fig. 3:**
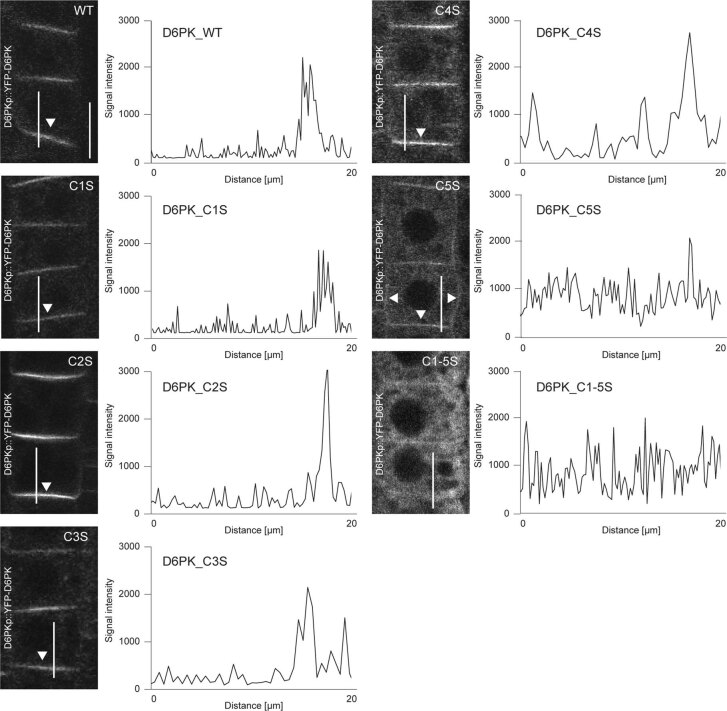
Profile blots confirm the reduced plasma membrane polarity of YFP-D6PK_C4S, C5S and C1-5S. Representative confocal images as shown in Fig. 2A (left panels) and profile blots (right panel) over a 20 µm line, as shown in the confocal images, confirm the reduction in relative plasma membrane protein abundance of YFP-D6PK_C4S, C5S and C1-5S. Images are representative of results from at least three independent experiments, imaging at least five roots each time.

To examine the role of the CXX(X)P motifs, we analysed mutants of yellow fluorescent protein (YFP)-tagged D6PK (YFP–D6PK) where cysteines (C) were replaced by the sterically similar serines (S), commonly used to replace C^19–21^. In turn, P2 to P5 were replaced by glycines (G). When transiently overexpressed in protoplasts or when stably expressed from a *D6PK* promoter fragment (D6PKp::YFP–D6PK) in a *d6pk d6pkl1* mutant, variants carrying C1S and C2S single-cysteine mutations showed a cellular distribution similar to wild-type D6PK (Fig. 2a–c and Extended Data Figs. 2a and 3). A gradual relative increase in the cytosolic abundance of the proteins became apparent in the case of the C4S and C5S variants, as well as in C1-5S, where subsequently all five Cs had been mutated (Fig. 2a–c and Extended Data Fig. 3). In YFP–D6PK C5S and C1-5S, plasma membrane signal and YFP–D6PK polarity were reduced, not only as a consequence of a relative signal decrease at the basal plasma membrane but also as a consequence of a signal increase at the lateral membrane (Fig. 2a,c). In turn, P2G through P5G as well as P2-5G mutations did not result in any apparent effect on YFP–D6PK distribution (Extended Data Fig. 2b). Beyond normal experiment-to-experiment variation, the auto- and trans-phosphorylation activities of D6PK were not affected in any of the variants (Extended Data Fig. 2c). The effects of the alanine replacement mutants YFP–D6PK C1A or C5A had effects comparable to those of YFP–D6PK C1S and C5S, respectively, suggesting that the choice of mutant amino acids had no unintended side effects (Extended Data Fig. 2a).

Rapid YFP–D6PK trafficking is dependent on the BFA-inhibited guanine nucleotide exchange factor GNOM^13^. When we treated seedlings with BFA, following a pre-treatment with the translation inhibitor cycloheximide (CHX) to prevent interference from de novo synthesized YFP–D6PK, we found that both the plasma membrane-localized YFP–D6PK and the largely cytosolic YFP–D6PK C1-5S responded very similarly, indicating that YFP–D6PK C1-5S was still subject to intracellular protein transport (Fig. 2d–i). Fluorescence recovery after photobleaching (FRAP) experiments revealed that plasma membrane-resident YFP–D6PK C5S, which was still detectable at the plasma membrane, had an increased mobility in the plasma membrane when compared to YFP–D6PK or YFP–D6PK C2S (Fig. 2j–l). We concluded that the cysteine residues of the CXX(X)P motifs, most prominently C4 and C5, were essential for D6PK plasma membrane association and required for the proper mobility of the protein in or towards the plasma membrane.

We next assessed whether the cysteine mutants complemented the phototropism defect of *d6pk d6pkl1* hypocotyl by exposing dark-grown seedlings to unilateral blue light (Fig. 2m,n)^9^. Whereas the wild type and the C1S to C3S transgenes efficiently rescued the *d6pk d6pkl1* phototropism defect, this ability was partially and fully impaired with the C4S, C5S and C1-5S transgenes (Fig. 2m,n). In cells of etiolated seedling hypocotyls, the intracellular abundance of YFP–D6PK C1-5S was increased, and its polar distribution reduced when compared to YFP–D6PK (Fig. 2o–q). The differential localization correlated with the inability of YFP–D6PK C1-5S to complement the apical hook formation defect and the reduced auxin transport in dark-grown *d6pk d6pkl1* mutant hypocotyls when compared to mutants complemented with YFP–D6PK (Fig. 2r and Extended Data Fig. 2d).

### Cysteines of the middle domain are not redox sensitive

Cysteines are highly reactive amino acids that can stabilize proteins through disulfide bonds^22,23^. In our analysis for redox sensitivity, we identified D6PK cysteines C183 and C189, but none of the five CXX(X)P motif cysteines, as being redox sensitive. Their remarkably low labelling efficiency in comparison to that of most other D6PK cysteines may be indicative of an inefficient penetration of the modifying reagents or the presence of other cysteine modifications (Supplementary Data Fig. 2a)^24^.

### The middle domain does not contribute to metal binding

Multiple cysteine residues may cooperate to form cation or cation-containing ligand complexes, such as Fe–S clusters^22^. We assessed the ability of D6PK to engage in interactions with Ca^2+^, Mg^2+^, Fe^2+^ or Zn^2+^ using recombinant GST-tagged GST–D6PK, GST–D6PKΔMID, a middle domain deletion variant and GST–D6PK_MID for the expression of the middle domain (Supplementary Data Fig. 2b,c). Although cation binding was detected in our ionometric analyses of GST–D6PK, the middle domain deletion in GST–D6PKΔMID did not affect this binding (Supplementary Data Fig. 2b). We therefore excluded the possibility that the middle domain engages in binding to the four cations tested.

### D6PK is an S-acylated protein

**Fig. 3:**
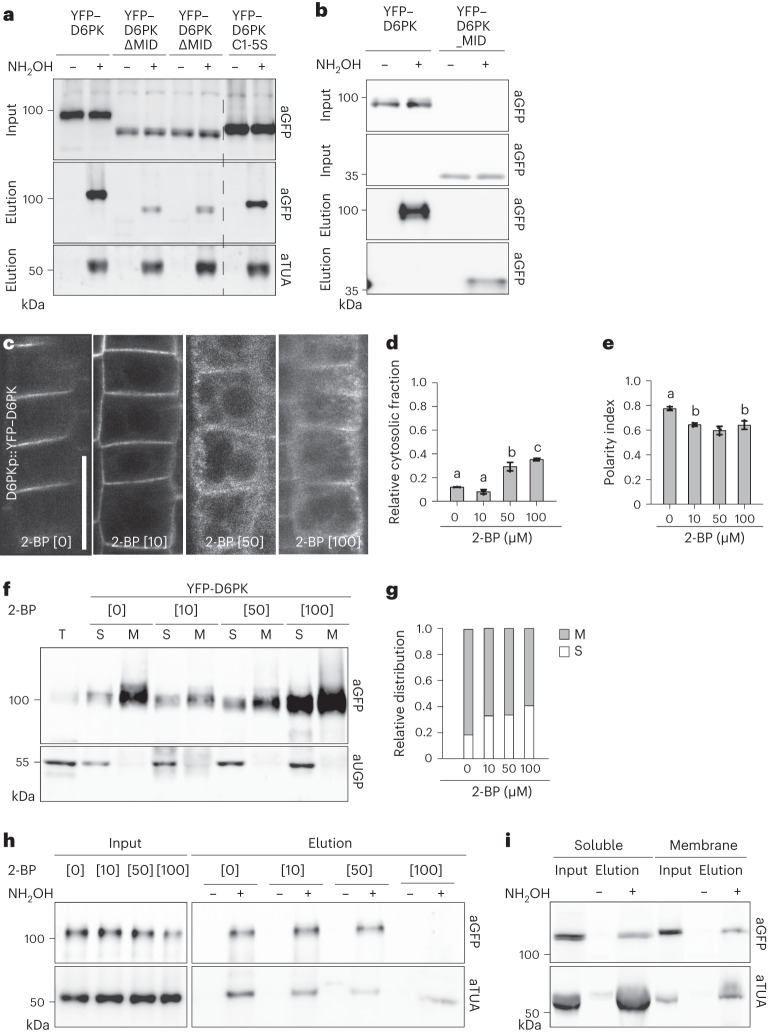
D6PK is S-acylated inside and outside of the middle domain. **a**,**b**, Immunoblots of total protein inputs and protein elutions after an acetyl biotin switch of protein extracts prepared from transgenic plants expressing YFP–D6PK wild type and variant proteins as specified (**a**) or YFP–D6PK wild type and YFP–D6PK_MID (**b**). Anti-GREEN FLUORESCENT PROTEIN (aGFP) recognizes the YFP-tag; aTUA recognizes the soluble palmitoylated tubulin A protein. **c**, Representative confocal images of root epidermal cells from 6-day-old stably transformed *A. thaliana* seedlings expressing the wild-type YFP–D6PK in *d6pk d6pkl1* after treatment with the palmitoylation inhibitor 2-BP. Scale bar, 20 µm. **d**,**e**, The means and standard deviations of the relative cytoplasmic fractions (**d**) and the polarity indices (**e**) as determined from confocal images as shown in **c**. The three data points represent the means of five measurements from one root, representing a biological replicate. **f**, Immunoblots of membrane (M) and soluble (S) fractions after fractionation of protein extracts from transgenic plants expressing YFP–D6PK after 2-BP treatment, as specified. T, total protein extract before fractionation. Anti-UDP-glucose pyrophosphorylase (aUGP) is a marker for the soluble fraction. **g**, The relative signal distribution between the membrane (M) and the soluble (S) fractions of the immunoblot shown in **f**. **h**,**i**, Immunoblots of total protein inputs and protein elutions after an acetyl biotin switch of protein extracts prepared from transgenic plants expressing wild type YFP–D6PK after 2-BP treatments (**h**) or after subcellular fractionation (**i**). In **d**, the statistically significant difference between groups was determined by one-way ANOVA, and means were compared using a Tukey’s test (**d**, *F*(3, 48) = 96.86, *P* < 0.0001; **e**, *F*(3, 48) = 25.80, *P* < 0.0001). Different letters indicate a statistically significant difference. All S-acylation assays and immunoblots were repeated at least twice with comparable results. Source data

Cysteine S-acylation, most prominently palmitoylation, is a common modification of membrane-associated proteins^25,26^. When we analysed S-acylation in seedlings expressing the respective transgenes, we detected S-acylation of full-length YFP–D6PK but also of YFP–D6PK_MID, YFP–D6PKΔMID and YFP–D6PK C1-5S, while only little YFP was recovered when purified from a line expressing untagged YFP (Fig. 3a,b and Supplementary Data Fig. 3)^27,28^. We concluded that YFP–D6PK is S-acylated in and outside of the middle domain.

After treatments with increasing concentrations of the palmitoylation inhibitor 2-bromo palmitate (2-BP), we observed a gradual increase in the relative cytoplasmic abundance of YFP–D6PK in root epidermal cells and a reduction in YFP–D6PK polarity (Fig. 3c–e). In immunoblots following cellular fractionation of 2-BP-treated seedlings, we detected a relative increase in YFP–D6PK solubility after 2-BP treatment but also an overall increase in YFP–D6PK abundance (Fig. 3f,g). In an acyl resin-assisted capture (acyl-RAC) assay with extracts from 2-BP-treated samples, S-acylation was lost at 2-BP concentrations that led to a loss of the protein from the plasma membrane (Fig. 3c,h). Thus, although 2-BP is known to affect multiple cellular responses and also trafficking events, 2-BP has effects on YFP–D6PK that are similar to those observed for the cysteine-mutated YFP–D6PK^29^.

**Extended Data Fig. 4:**
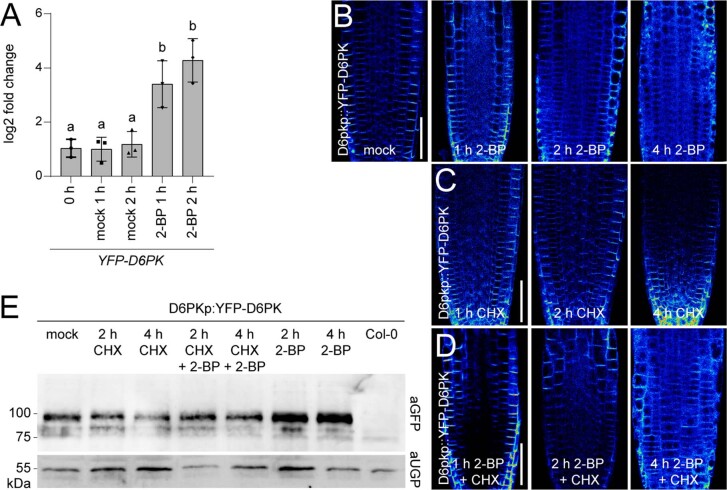
2-BP treatments lead to increased YFP-D6PK transcript abundance. **A**. Bar charts displaying the means and standard deviations of a qRT-PCR experiment testing for the transcription of YFP-D6PK after mock and 2-BP (2-bromo palmitate) treatment, as specified, from three biological replicates displayed as individual data points. Expression levels were compared using a One-way ANOVA F(4, 10) = 18.85, p = 0.0001. **B - D**. Representative confocal images of root tips from three days-old transgenic seedlings expressing D6PKp::YFP-D6PK and treated with 2-BP and CHX (cycloheximide) as specified. Scale bars = 50 µm. **E**. Immunoblots with anti-GFP (aGFP) ad anti-UGP (aUGP) antibodies on total protein extracts treated with 2-BP and CHX (cycloheximide) as specified. The CHX chase experiment, including the imaging and immunoblot, was performed once. Source data

When we compared S-acylation of YFP–D6PK before and after BFA treatment, we did not detect any apparent quantitative changes, leading us to conclude that dynamic changes in D6PK S-acylation unlikely occur during YFP–D6PK trafficking or its release from the plasma membrane (Fig. 3i)^30^. When we assessed the effects of 2-BP treatment on *YFP–D6PK* expressed from the *D6PK* promoter by quantitative real-time PCR (qRT-PCR), we observed an approximately fourfold increase in *YFP–D6PK* transcription after 2-BP treatment (Extended Data Fig. 4a). At the same time, assays with the protein synthesis inhibitor CHX, allowing to trace YFP–D6PK abundance in response to 2-BP treatments, did not lead to detectable changes in YFP–D6PK abundance (Extended Data Fig. 4b–e). Increased *YFP–D6PK* transcription, rather than increased YFP–D6PK protein stability, should thus be causal for the observed increase in YFP–D6PK abundance after 2-BP treatment, possibly part of a positive feedback mechanism.

### Phosphorylation at D6PK S310 and S311 is required for its proper plasma membrane trafficking and polarity

**Extended Data Fig. 5:**
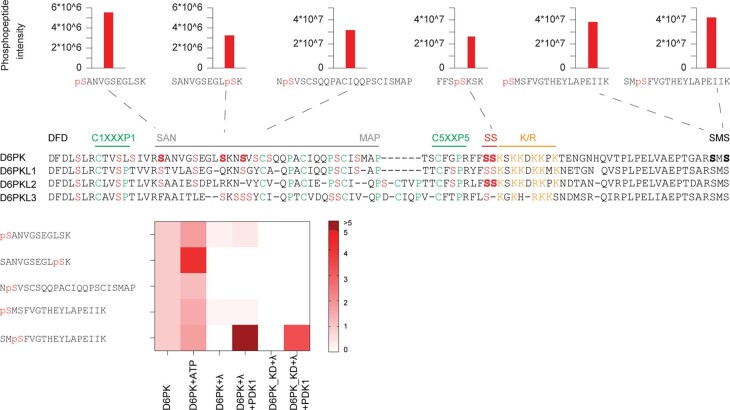
Mass spectrometric analysis of middle domain phosphosites. Muscle alignment of D6PK and D6PKL middle domain sequences with the 31 amino acids deleted in YFP-D6PK∆SAN marked by a line. Serine residues in the middle region are highlighted in red, serines detected in their phosphorylated form in *in vitro* phosphorylation experiments are shown in bold letters together with their respective phosphopeptide intensity blots (upper panels) for the specified tryptic peptide fragments. The heatmap shows the relative abundance of the phosphorylated peptide in the different samples allowing to differentiate auto- and transphosphorylation events. The peptide corresponding to S310/S311, FFSSKSK, was recovered very rarely and only in its phosphorylated form, rendering it impossible to establish the corresponding correlations.

In D6PK and many other AGC1 kinases, the CXX(X)P repeats and the polybasic K/R-rich motif are separated by putative serine phosphosites (Fig. 1a)^7^. In D6PK, these correspond to S310 and S311, and we identified D6PK S311 as phosphorylated after in vitro phosphorylation (Extended Data Fig. 5). The equivalent D6PKL2 S392 and S393 had been reported as phosphorylated serines in a thematically unrelated study (Extended Data Fig. 5)^31^.

**Fig. 4:**
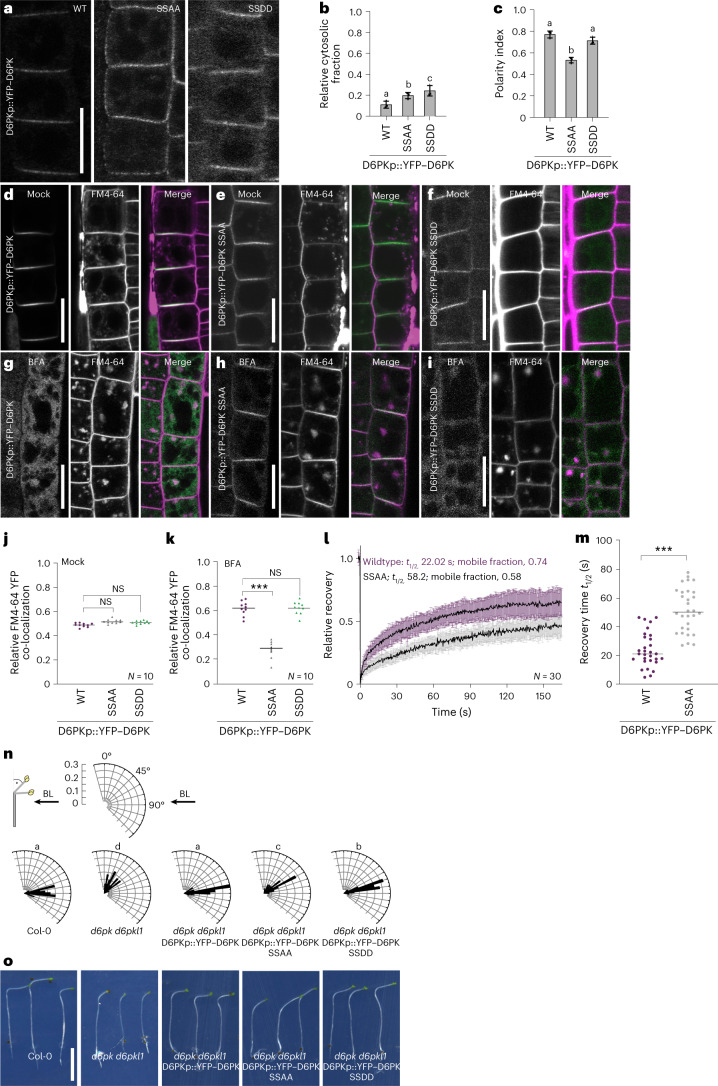
D6PK S310/S311 are serine phosphosites required for YFP–D6PK trafficking and polarity. **a**, Representative confocal images of root epidermal cells from 6-day-old seedlings expressing the wild-type YFP–D6PK and mutant SSAA and SSDD in *d6pk d6pkl1*. Scale bar, 20 µm. **b**,**c**, The means and standard deviations of the relative cytoplasmic fractions (**b**) and the polarity indices (**c**) as determined from images as shown in **a**. The three data points represent the means of five measurements from one root, representing a biological replicate. **d**–**i**, Representative confocal images of root epidermal cells from 5-day-old seedlings expressing D6PKp::YFP–D6PK (**d**), D6PKp::YFP–D6PK SSAA (**e**) and D6PKp::YFP–D6PK SSDD (**f**) in *d6pk d6pkl1* after mock treatment and FM4-64 staining or of D6PKp::YFP–D6PK (**g**), D6PKp::YFP–D6PK SSAA (**h**) and D6PKp::YFP–D6PK SSDD (**i**) after BFA (10 µM for 15 min; 1 h 50 µM CHX pre-treatment) and FM4-64 staining. Scale bars, 20 µm. **j**,**k**, Graphs with quantifications of YFP–D6PK and FM4-64 signals after mock (**j**) and BFA treatment (**k**) from images as shown in **d**–**i** of 15 cells from at least three seedlings. The imaging data are representative for at least three independent experiments. **l**,**m**, Results from FRAP experiments performed with epidermal cells of 5-day-old seedlings. Shown are the means and standard deviations of normalized recovery curves (**l**) and individual recovery times *t*_1/2_ (**m**) at the basal plasma membrane for wild-type YFP–D6PK and mutant SSAA. **n**, Rose diagrams with the frequency distribution in 5° intervals of hypocotyl bending angles of ≥60 three-day-old dark-grown seedlings, as specified, after unilateral BL exposure (arrow) for 4 h. **o**, Photographs of three representative seedlings from the experiment validated in **n**. Scale bar, 0.5 cm. In **b** and **n**, the statistically significant difference between groups was determined by one-way ANOVA (**b**,**c**,**n**), and means were compared using a Tukey’s test (**b**, *F*(2, 36) = 56.43, *P* < 0.0001; **c**, *F*(2, 36) = 24.63, *P* < 0.0001; **n**, *F*(4, 295) = 176.0, *P* < 0.0001). Different letters indicate a statistically significant difference. Two-tailed Student’s *t*-test (**j**,**k**,**m**); NS, not significant; ***P < 0.001.

**Extended Data Fig. 6:**
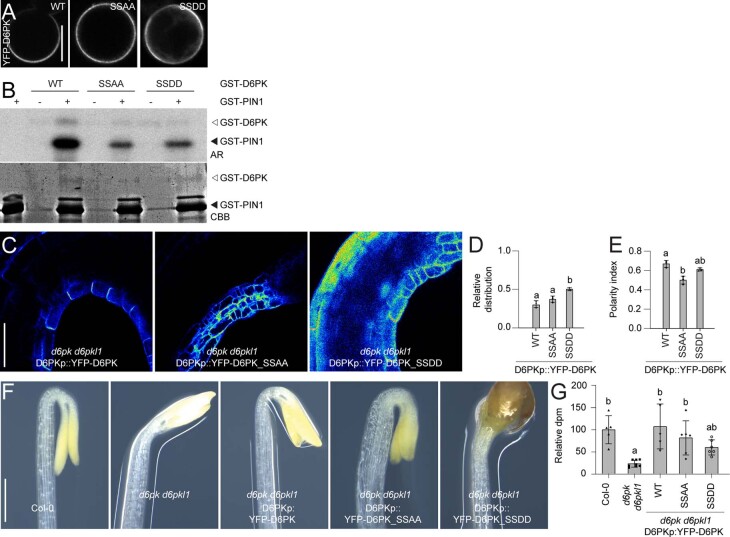
D6PK_SSAA mutations impair D6PK polar distribution and compromise basipetal auxin transport. **A** Representative confocal images of transiently transformed protoplasts overexpressing wild type YFP-D6PK from 35Sp::YFP-D6PK or its mutant variants with SSAA and SSDD mutations. Scale bar = 10 µm. Transformations were performed twice and multiple protoplasts were imaged each time yielding comparable results. **B**. Result from *in vitro* kinase assays with recombinant wild type and mutant GST-D6PK as specified using a cytoplasmic loop fragment of PIN1 in GST-PIN1 as trans-phosphorylation substrate. AR, autoradiography; CBB, Coomassie Brilliant Blue-stained gel as loading control. Kinase assays were performed twice and similar results were obtained. **C**. Representative confocal images of the apical hook region of transgenic seedlings expressing the transgenes as specified in the *d6pk d6pkl1* background. **D** and **E**. Bar charts displaying the means and standard deviations of the relative cytoplasmic fractions (D) and the polarity indices (E) as determined from confocal images as shown in (C). The three data points represent the means of five measurements from one root, representing a biological replicate. **F**. Representative images of three-days old dark-grown seedlings with the specified genotypes. Scale bar = 0.5 mm. **G**. Graph displaying the mean and standard deviation of basipetal auxin transport as measured in four-days old dark-grown seedlings with the specified genotypes. Each data point represents a measurement taken from five seedlings. In (D), (E) and (G), the statistically significant difference between groups was determined by One-way ANOVA and means were compared using a Tukey’s test (D, F(2, 36) = 18.80, p = 0.0000026; E, F(2, 36) = 15. 79, p = 0.0000119; G, F(4,25) = 7.997, p = 0.00027). Different letters indicate a statistically significant difference.

To examine the importance of S310 and S311 for D6PK function, we expressed the phosphorylation-impaired variant YFP–D6PK_SSAA (SSAA) and the putative phosphorylation-mimicking YFP–D6PK_SSDD (SSDD) in *d6pk d6pkl1* (Fig. 4a). Mutagenesis of S310 and S311 resulted in an increased intracellular accumulation of the protein in the case of SSAA that was even more pronounced in the case of SSDD (Fig. 4b). Moreover, SSAA showed an increased apolar distribution at the plasma membrane when compared to YFP–D6PK and the SSDD variant, which may lead to an absolute decrease of SSAA at the basal plasma membrane, resulting in the observed relative increase in intracellular SSAA levels (Fig. 4c and Extended Data Fig. 6). Importantly, after BFA treatment, SSAA was less efficiently internalized than YFP–D6PK or SSDD (Fig. 4d–k). Furthermore, SSAA showed a slower recovery and was reduced in the mobile fraction when compared with YFP–D6PK, so that we concluded that S310/S311 are important for efficient YFP–D6PK trafficking (Fig. 4l,m).

The importance of S310/S311 for proper YFP–D6PK function was also supported by our observation that SSAA only partially complemented the hypocotyl phototropism defect of *d6pk d6pkl1* (Fig. 4n,o). Both SSAA and SSDD showed an increased cytosolic abundance in hypocotyl cells and, in the case of SSAA, a strong impairment of YFP–D6PK polarity, which was accompanied by an apparently disturbed tissue patterning (Extended Data Fig. 6c–e). Regardless, SSAA was more efficient than SSDD in rescuing the apical hook formation defect of *d6pk d6pkl1*. This behaviour correlated with the, in comparison to SSDD, stronger capacity of SSAA in promoting auxin transport in hypocotyls (Extended Data Fig. 6f,g). The latter may be explained by the fact that SSAA, due to its longer residence time at the plasma membrane, may activate auxin transport at the plasma membrane for longer (Fig. 4k). We concluded that D6PK S310/S311 are required for proper YFP–D6PK trafficking and polarity regulation. As we consistently detected a reduction in PIN1 trans-phosphorylation by GST–D6PK_SSAA or GST–D6PK_SSDD in comparison to GST–D6PK, we cannot exclude the possibility that the differential effects in planta are the result of this reduced kinase activity rather than of its altered cell biological behaviour (Extended Data Fig. 6b).

### D6PK phospho-S310/S311 accumulates intracellularly

**Fig. 5:**
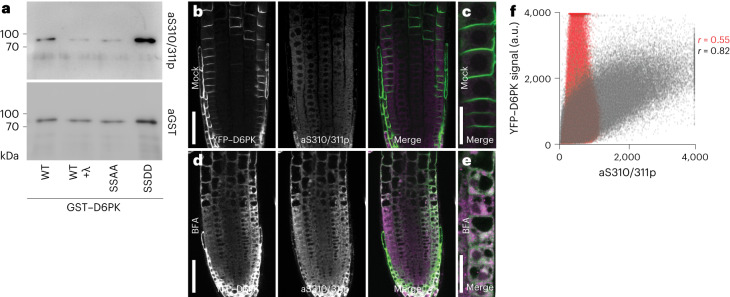
D6PK phosphorylated at S310/S311 accumulates intracellularly. **a**, Immunoblots with the phosphosite-specific D6PK aS310p/311p antibody and anti-GST (aGST; loading control) against recombinant wild-type GST–D6PK (WT) before and after λ-phosphatase treatment, as well as the S310/S311 phosphosite mutant GST–D6PK_SSAA and the phosphorylation-mimicking GST–D6PK_SSDD. Note that GST–D6PK is recovered as phosphorylated protein when expressed in and purified from bacteria. Purifications and immunoblots were performed twice with comparable results. **b**–**e**, Representative confocal images after immunostaining with aS310p/311p of 4-day-old *A. thaliana* seedlings overexpressing wild-type YFP–D6PK under mock conditions (**b**) and after BFA (10 µM for 30 min) treatment (**d**) and magnified confocal images of the epidermal cell layer of wild type YFP–D6PK under mock conditions (**c**) and after BFA (10 µM for 30 min) treatment (**e**), as shown in **b** and **d**. Scale bars, 50 µm (**b**,**d**) and 20 µm (**c**,**e**). **f**, The quantification of the co-localization between YFP–D6PK and aS310p/S311p after BFA treatment (grey) and a corresponding mock treatment (red) as shown in **b**–**e**. Note that the Cy3 signal (aS310p/311p) shows co-localization (*r* = 0.82) with YFP–D6PK only after BFA treatment (**d**,**e**) but not when mock-treated (*r* = 0.55; **b**,**c**). Immunostainings were carried out three independent times with comparable results. a.u., arbitrary units.

To evaluate YFP–D6PK S310/S311 phosphorylation by immunostaining, we generated aS310p/311p using a phosphorylated peptide epitope conserved between D6PK, D6PKL1 and D6PKL2 (Fig. 5a). While aS310p/311p recognized recombinant GST–D6PK, its affinity was strongly reduced after dephosphorylation with λ-phosphatase, indicating that the protein was phosphorylated when recovered from the bacterial host (Fig. 5a). aS310p/311p efficiently recognized the phosphorylation mimicking GST–D6PK_SSDD variant but not GST–D6PK_SSAA (Fig. 5a).

**Extended Data Fig. 7:**
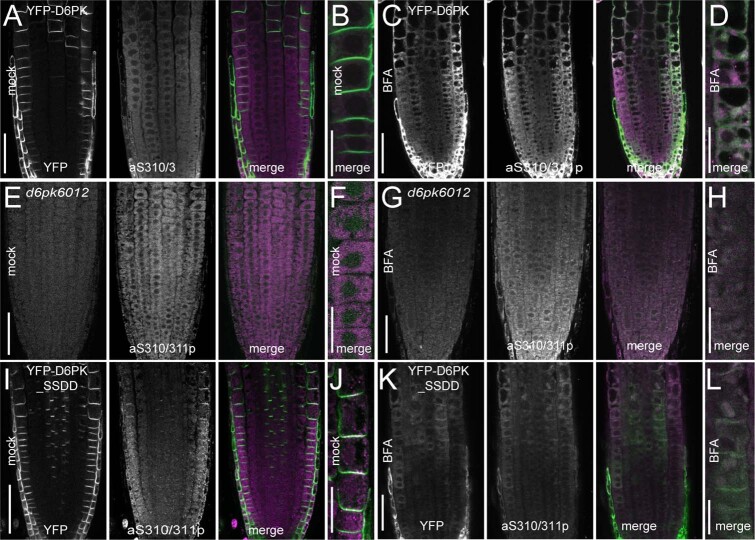
D6PK phosphorylated at S310/S311 accumulates intracellularly. **A - L**. Representative confocal images after immunostaining with aS310p/311p of four-days-old *Arabidopsis thaliana* seedlings overexpressing wild type YFP-D6PK (A -D) or YFP-D6PK_SSDD (I – L) under mock conditions and after BFA treatment. The *d6pk d6pkl1 d6pkl2* (*d6pk012*) triple mutant is included for comparisons. Images shown in A – D are the same images as those shown in Fig. 5B - E. Scale bars = 50 µm (A, C, E, G, I, K) and 20 µm (B, D, F, H, J, L). Images are representative images taken from multiple roots imaged from at least two independent experiments.

In immunostaining, aS310p/311p did not detect YFP–D6PK at the plasma membrane in untreated cells but detected internalized YFP–D6PK after BFA treatment in the cytosol (Fig. 5b–f). D6PK S310/S311 phosphorylation may thus be predominantly associated with internalized D6PK. The specificity of the signal was verified in the *d6pk d6pkl1 d6pkl2* mutant, where we did not observe an increase in the cytosolic signal intensity after BFA treatment (Extended Data Fig. 7a–h). Furthermore, aS310p/S311p recognized SSDD also at the plasma membrane because the S310/S311 phosphorylation is irreversibly mimicked (Extended Data Fig. 7i–l). S310/S311 phosphorylation could thus be part of the mechanism underlying the dissociation of YFP–D6PK from the plasma membrane, its internalization and trafficking. This conclusion is additionally supported by our observation that SSAA has reduced polar distribution and an extended residence time at the plasma membrane compared to YFP–D6PK (Fig. 4l,m).

### YFP–D6PK polar distribution is impaired in YFP–D6PKΔSAN

**Fig. 6:**
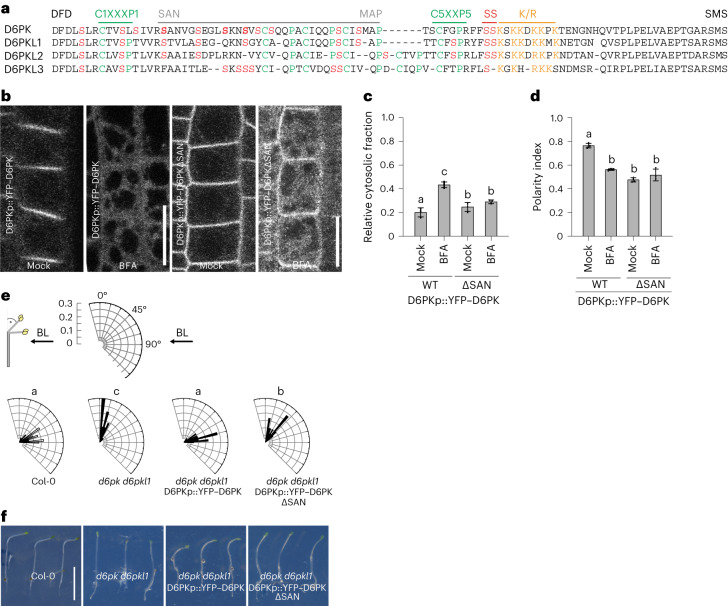
YFP–D6PK polar plasma membrane distribution is impaired in YFP–D6PKΔSAN. **a**, Muscle alignment of D6PK and D6PKL middle domain sequences with the 31 amino acids deleted in YFP–D6PK∆SAN marked by a line. Serine residues in the middle region are highlighted in red, including residues previously found phosphorylated in in vitro phosphorylation experiments (bold). See also Extended Data Fig. 8. **b**, Representative confocal microscopy images of root epidermal cells from 6-day-old stably transformed *A. thaliana* seedlings expressing the wild-type YFP–D6PK and mutant YFP–D6PK∆SAN after BFA (50 µM for 15 min; 1 h 50 µM CHX pre-treatment) or a corresponding mock treatment in *d6pk d6pkl1*. Scale bars, 20 µm. **c**,**d**, The means and standard deviations of the relative cytoplasmic fractions (**c**) and the polarity indices (**d**) as determined from confocal images as shown in **b**. The three data points represent the means of five measurements from one root, representing a biological replicate. **e**, Rose diagrams with the frequency distribution in 5° intervals of hypocotyl bending angles of ≥30 three-day-old dark-grown seedlings as specified after unilateral BL exposure for 4 h. The arrow indicates the direction of the BL exposure. **f**, Photographs of three representative seedlings from the experiment validated in **e**. Scale bar, 0.5 cm. In **c** and **d**, the statistically significant difference between groups was determined by one-way ANOVA, and means were compared using a Tukey’s test (**c**, *F*(3, 48) = 111.1, *P* < 0.0001; **d**, *F*(3, 48) = 43.44, *P* < 0.0001). Different letters indicate a statistically significant difference.

**Extended Data Fig. 8:**
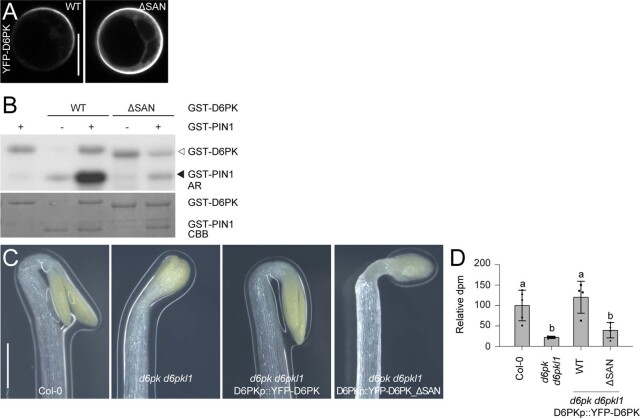
The D6PK_ΔSAN mutation impairs D6PK polar distribution and compromises basipetal auxin transport. **A** Representative confocal images of transiently transformed protoplasts overexpressing wild type YFP-D6PK from 35Sp::YFP-D6PK or its mutant variants with the ΔSAN deletion. Scale bar = 10 µm. **B**. Result from *in vitro* kinase assays with recombinant wild type and mutant GST-D6PK as specified using a cytoplasmic loop fragment of PIN1 in GST-PIN1 as trans-phosphorylation substrate. AR, autoradiography; CBB, Coomassie Brilliant Blue-stained gel as loading control. Localization and phosphorylation data are representative of two independent experiments. **C**. Representative images of three-days old dark-grown seedlings with the specified genotypes. Scale bar = 0.5 mm. **D**. Bar chart displaying the mean and standard deviation of relative basipetal auxin transport as measured in four-days old dark-grown seedlings with the specified genotypes. Each data point represents a measurement taken from five seedlings. In (D), the statistically significant difference between groups was determined by One-way ANOVA and means were compared using a Tukey’s test (D, F(3, 13) = 10.54, p = 0.0009).

Besides D6PK S310/S311, the middle domains of D6PK and D6PKLs contain a disproportionally high number of serine (and threonine) residues, some of which had been identified as being phosphorylated after mass spectrometric analysis of recombinant kinase-active D6PK (Fig. 6a and Extended Data Fig. 5). We examined the importance of these residues with lines expressing YFP–D6PKΔSAN, a deletion spanning 31 amino acids from the residues serine-alanine-asparagin (SAN) to methionine-alanine-proline (MAP), including eight serines but retaining the highly conserved C1XXXP1 and the functionally important C5XXP5 motifs, S310/S311, as well as the polybasic motif (Fig. 6a). YFP–D6PKΔSAN accumulated at the plasma membrane but showed a strongly decreased polar accumulation and was partially BFA insensitive when compared to the wild type (Fig. 6b–d and Extended Data Fig. 8a).

YFP–D6PKΔSAN, only to a minor extent, suppressed the phototropism and apical hook formation defects of *d6pk d6pk1*, which was in agreement with the inability of the YFP–D6PKΔSAN transgene to suppress the auxin transport in the *d6pk d6pk1* mutant (Fig. 6e,f and Extended Data Fig. 8). The very low signal intensities observed in lines expressing the transgene prevented us from analysing protein distribution in hypocotyl cells. We concluded that the deleted region, which included several putative phosphosites, was dispensable for plasma membrane association of the protein but required for maintaining its polar plasma membrane distribution and its BFA-sensitive trafficking. In in vitro phosphorylation experiments, GST–D6PKΔSAN was less active towards the PIN substrate than wild-type GST–D6PK, and this reduced phosphorylation activity may provide an alternative explanation for the observed biological effects, as it had already been reasoned for GST–D6PK_SSAA and GST–D6PK_SSDD (Extended Data Fig. 8b).

### PDK-dependent YFP–D6PK polarity and BFA-sensitive trafficking

**Fig. 7:**
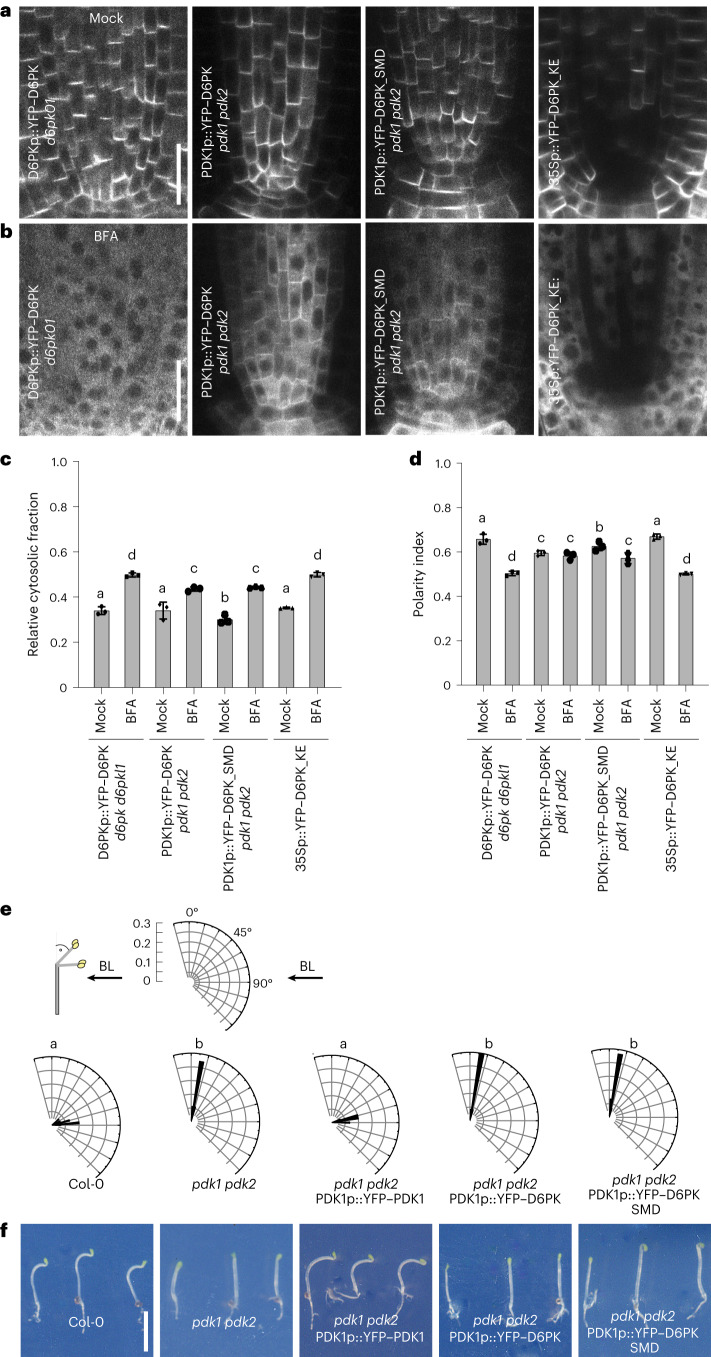
D6PK BFA sensitivity is altered in the *pdk1 pdk2* background. **a**,**b**, Representative confocal images of the stele of 5-day-old *A. thaliana* seedlings expressing YFP–D6PK, YFP–D6PK_SMD or YFP–D6PK_KE in the described genetic backgrounds under mock conditions (**a**) and after BFA (50 µM for 15 min) treatment (**b**). Scale bars, 20 µm. **c**,**d**, The means and standard deviations of the relative cytoplasmic fractions (**c**) and the polarity indices (**d**) as determined from confocal images as shown in **a** and **b**. The three data points represent the means of five measurements from one root, representing a biological replicate. **e**, Rose diagrams with the frequency distribution in 5° intervals of >10 hypocotyl bending angles of 3-day-old dark-grown seedlings as specified after unilateral BL exposure for 4 h. The arrow indicates the direction of the BL exposure. **f**, Photographs of three representative seedlings from the experiment validated in **e**. Scale bar, 0.5 cm. In **c**–**e**, the statistically significant difference between groups was determined by one-way ANOVA (**c**,**d**,**e**), and means were compared using a Tukey’s test (**c**, *F*(7, 96) = 99.11, *P* < 0.0001; **d**, *F*(7, 96) = 53.22, *P* < 0.0001; **e**, *F*(4, 39) = 279.5, *P* < 0.0001). Different letters indicate a statistically significant difference.

PDK1 and PDK2 activate D6PK and the related PAX through phosphorylation at their SMS activation loop^15,16^. When we examined YFP–D6PK, expressed from a *PDK1* promoter fragment, in the *pdk1 pdk2* double mutant, we found that YFP–D6PK was less polarly distributed in the plasma membrane of *pdk1 pdk2* than in the wild type (Fig. 7a–d). Due to the expression pattern of *PDK1*, this analysis had to be performed in root stele cells, where YFP–D6PK polarity is generally less pronounced than in root epidermal cells, which had been quantified in our other analyses. Trafficking of YFP–D6PK in *pdk1 pdk2* was at least partially BFA insensitive when compared to the wild type (Fig. 7a–d). At the same time, YFP–D6PK expressed from PDK1p::YFP–D6PK in *pdk1 pdk2* was not detected by aS310p/311p in epidermis or cortex cells after BFA treatment but only when expressing YFP–PDK1 from PDK1p::YFP–PDK1 (Extended Data Fig. 10).

As the effect of the activation loop phosphorylation-mimicking mutation in YFP–D6PK_SMD in *pdk1 pdk2* was similar to the behaviour observed with wild-type YFP–D6PK in *pdk1 pdk2*, we reasoned that the roles of PDK1 in regulating D6PK activity and D6PK trafficking are independent of each other (Fig. 7a–d)^15^. At the same time, the behaviour of the kinase-dead variant YFP–D6PK_KE was indistinguishable from that of wild-type YFP–D6PK, inviting the conclusion that the observed effects do not require D6PK activity but are controlled by an upstream regulator, with the PDKs or a protein kinase acting downstream from the PDKs being suitable candidates (Fig. 7a–d).

**Extended Data Fig. 9:**
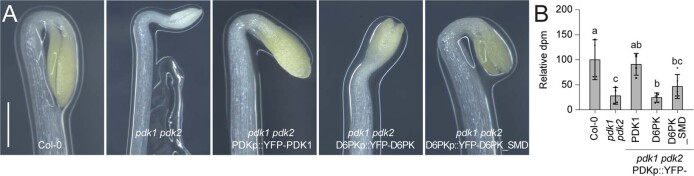
A D6PK activation loop mimicking SMD mutation is sufficient to suppress the apical hook formation and, partially, also the auxin transport defect of *pdk*1 pdk2 mutants. **A**. Representative images of three-days old dark-grown seedlings with the specified genotypes. Scale bar = 0.5 mm. **B**. Graph displaying the mean and standard deviation of relative basipetal auxin transport as measured in four-days old dark-grown seedlings with the specified genotypes. Each data point represents a measurement taken from five seedlings. In (B), the statistically significant difference between groups was determined by One-way ANOVA and means were compared using a Tukey’s test (B, F(4, 17) = 9.344, p = 0.0003). Different letters indicate a statistically significant difference.

**Extended Data Fig. 10:**
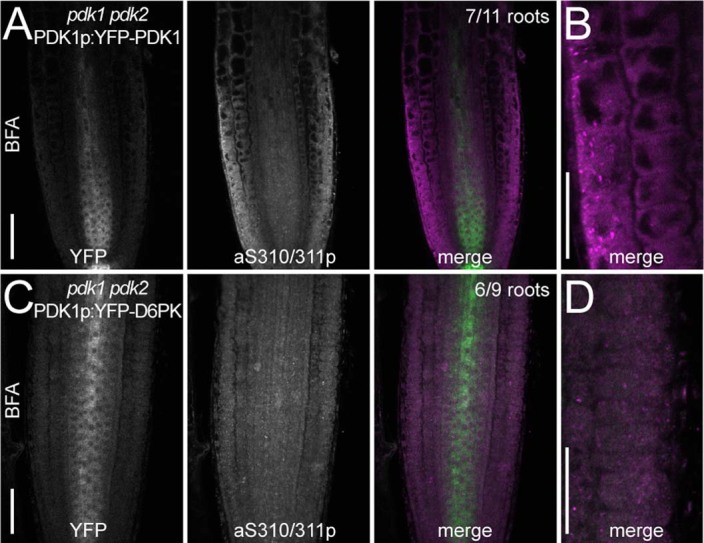
Immunostaining in the *pdk1 pdk2* complementation lines reveals D6PK activation loop phosphorylation after BFA treatment. **A** - **D**. Representative confocal images of roots (A, C) and magnifications of root cortex and epidermal cells (B, D) from four-days-old seedlings expressing YFP-PDK1 and YFP-D6PK (negative control) from the PDK1 promoter (*PDK1p*) after BFA treatment. Shown are YFP fluorescence, fluorescence after immunostaining with the aS310p/S311 antibody, as well as a merge of the images (A, C) and their magnification (B, D). Scale bars = 50 µm (A, C) and 20 µm (B, D). Immunostainings were performed twice with comparable results.

Importantly, and in line with the differential auxin transport activities measured in the respective seedling hypocotyls, the phototropism defect of the *pdk1 pdk2* mutant could be rescued by expression of YFP–PDK1 but not by the expression of YFP–D6PK or YFP–D6PK_SMD expressed from the *PDK1* promoter (Fig. 7e,f and Extended Data Fig. 9b). In *pdk1 pdk2* YFP–D6PK_SMD, the loss of *PDK* function is compensated by the SMD mutation in D6PK, which renders the kinase constitutively active, as previously reported^15^. Importantly, the activity and thus the functionality of the mutation in YFP–D6PK_SMD became apparent in our studies because YFP–D6PK_SMD, but not YFP–D6PK, complemented the apical hook formation defect of *pdk1 pdk2* when expressed from the *PDK1* promoter (Extended Data Fig. 9a). Our findings support the conclusion that YFP–D6PK intracellular trafficking depends on regulation by PDK kinases or kinases downstream from PDK1 but is independent of D6PK kinase activity.

## Discussion

D6PK is a polarly localized PIN-regulatory kinase that activates PIN-mediated cell-to-cell auxin transport and thereby controls developmental processes and tropic responses^5,8–10^. We previously showed that the middle domain of D6PK is required and sufficient to guide D6PK to the basal (root tip-directed) plasma membrane and identified a polybasic K/R motif as a phosphoinositide-interacting motif required for D6PK plasma membrane association^13^. Here we show that cysteine acylation of CXX(X)P motifs and phosphorylation at S310/S311, and possibly other phosphosites, are functionally important and in the case of phosphoregulation required for proper D6PK internalization. We found that, besides the CXX(X)P motif cysteines, also cysteines outside of the middle domain are subject to this modification. Mutation of the CXX(X)P motif cysteines or acylation inhibition with 2-BP affected plasma membrane localization and increased the amount of soluble protein, indicating that acylation is required for membrane-anchoring of D6PK. We could, however, not obtain any evidence for a dynamic acylation modification of D6PK. While this work was in progress, an untargeted proteome-wide study searching for S-acylated proteins identified one CXX(X)P motif cysteine from D6PK, D6PKL1 and AGC1.9 as being S-acylated, providing independent support for our experimental finding^32^.

In turn, we revealed that D6PK S310/S311 phosphorylation is predominantly detected when the protein accumulates intracellularly after inhibition of the BFA-sensitive GEF GNOM. Mutant variants of D6PK S310/S311 or those lacking adjacent putative phosphorylation sites, deleted in the D6PKΔSAN variant, do not accumulate intracellularly after GNOM inhibition. This suggests that D6PK phosphorylation at these sites may be a requirement for D6PK endocytosis. At the same time, these variants show decreased polar distribution at the plasma membrane, indicating that their reduced plasma membrane turnover promotes their increased lateral diffusion.

Essentially all D6PK mutant variants with D6PK distribution or trafficking were also severely impaired in their ability to complement the *d6pk d6pkl1* defects in phototropic hypocotyl bending, apical hook formation and basipetal auxin transport through the dark-grown seedling hypocotyl^9^. A single interesting exception was the SSAA variant, whose expression rescued partially the phototropism defect and fully the apical hook formation defect of *d6pk d6pk1* but, at the same time, strongly altered the cellular organization of the hypocotyl and strongly complemented the auxin transport defect of *d6pk d6pk1*. These differential effects may be explained by the increased residence time of SSAA at the plasma membrane where the protein may be relatively more efficient in activating PIN auxin transporters.

We further identify PDKs as candidate kinases that, directly or indirectly, promote D6PK internalization. PDKs may thus activate D6PK through activation loop phosphorylation, as previously shown^15^. In addition, we identify several additional phosphorylation sites in the middle domain in the adjacent region that are auto-phosphorylated by D6PK and that we deleted in D6PKΔSAN. It is intriguing that the processes of kinase activation, phosphorylation and internalization are coupled, and this may constitute an important process for kinase trafficking, on the one side, and PIN activity regulation, on the other. It is intriguing to speculate that the negative charges introduced through these phosphorylations impair the interactions of the positively charged K/R-rich motif with negatively charged membrane phospholipids.

**Fig. 8:**
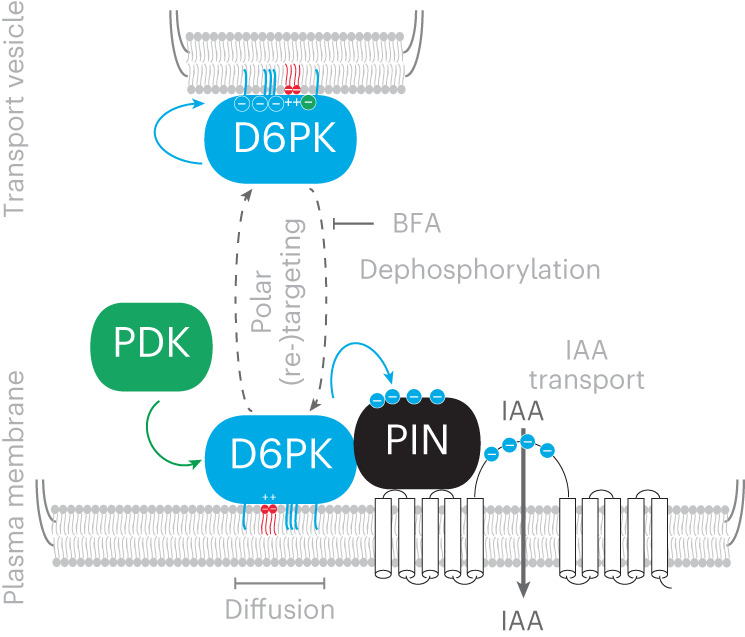
Model of the proposed PDK-dependent phosphoregulation of D6PK. Schematic representation of PDK- and D6PK-dependent phosphorylation events during D6PK transport to and from the plasma membrane. PDK has a dual function in activating D6PK through SMS motif phosphorylation and in promoting D6PK endocytosis through S310/S311 phosphorylation. D6PK auto-phosphorylates D6PK phosphosites deleted in the D6PKΔSAN region. Colour code for plasma and vesicle membrane elements: red, phospholipids; blue, S-acylations. Encircled minus symbols denote phosphorylations at the respective proteins, the colour code reflects the identity of the upstream regulatory protein kinase. Plus symbols denote positively charged amino acids of the K/R motif. IAA, indole-3-acetic acid, auxin. The identity of the dephosphorylating phosphatase is unknown.

Remarkably, the sequence elements characterized here are present in most, if not all, AGC1 kinases, and their interplay, as proposed here for D6PK, may also be relevant for the regulation of their polarity and activity (Extended Data Fig. 1). Intriguingly, these elements are not conserved in the AGC3 kinases PINOID, WAG1 or WAG2, which are non-polar plasma membrane resident kinases whose trafficking is largely unaffected by BFA (Extended Data Fig. 1). The non-polar distribution of these AGC3 kinases could thus be the consequence of differences in the middle domain, such as the absence of essential phosphorylation sites. In summary, we reveal a PDK- and phosphorylation-dependent mechanism for the regulation of D6PK polarity and turnover that may also explain the polarity regulation of other AGC1 kinases and, indirectly, the altered polarity and transporting properties of AGC3 kinases (Fig. 8).

## Methods

### Biological material

All experiments were conducted in wild-type *A. thaliana* (Columbia-0, Col-0) or previously published *d6pk01* (*d6pk d6pk-like1*), *d6pk012* (*d6pk d6pk-like1 d6pk-like2*) and *pdk1-14 pdk2-4* (*pdk1 pdk2*) mutants in the Col-0 background^8,16^. Transgenic lines expressing YFP–D6PK, YFP–D6PKΔMID with a middle domain deletion, or YFP–D6PK_MID with the middle domain alone from the 35S CaMV (35Sp) or a D6PK promoter (*D6PKp*) fragment in *d6pk d6pkl1* were previously described^13^. The transgenic lines expressing kinase-dead 35S::YFP–D6PK KE and 35Sp::YFP were previously published^8^, as well as a line expressing PDK1p:YFP–PDK1 in *pdk1 pdk2*^16^.

### Sequence alignments

For protein alignments, protein sequences of AGC kinases from *A. thaliana* and other angiosperms, as specified, were retrieved from PLAZA4.0 and aligned using the Muscle Alignment tool of the Geneious R11.1.3 software^33^. The alignments were then manually adjusted to achieve an optimal alignment of the CXX(X)P motifs, the K/R-rich motif and the serine residues.

### Cloning procedures

For site-directed mutagenesis, previously described constructs for the expression of *D6PK* in plants, D6PKp::YFP–D6PK or 35Sp::YFP–D6PK, or in *Escherichia coli*, GST–D6PK, were mutagenized with oligonucleotides using established protocols (Supplementary Table S1)^8,34^. To generate YFP–D6PKΔSAN_MAP, amino acids 268–300 were deleted from the previously published D6PKp::YFP–D6PK, 35Sp::YFP–D6PK and GST–D6PK using a PCR-based published procedure (Supplementary Table S1)^35^. To generate GST–GNC, *GNC* (*GATA*, *NITRATE-INDUCIBLE*, *CARBON-METABOLISM INVOLVED*) complementary DNA was PCR-amplified using a pair of oligonucleotides that introduced *EcoRI* and *SalI* restriction sites and cloned into the expression vector pGEX-6P-1 (Cytiva). To obtain PDK1p::YFP–D6PK and PDK1p::YFP–D6PK_SMD, D6PK was cloned into pDONR207 by Gateway BP cloning (Invitrogen). The entry vector was mutated by site-directed mutagenesis at the activation loop D6PK serine S345 to obtain the activation loop phosphorylation mimicking D6PK_SMD. Wild-type D6PK and D6PK_SMD were then subcloned into pART27-pPDK1::YFP::GWR using Gateway LR cloning to obtain PDK1p::YFP–D6PK and PDK1p::YFP–D6PK_SMD^16^.

### Protoplast transformation

Protoplasts were obtained by digestion of cells from an *A. thaliana* root cell culture as previously described^36^. The protoplasts were then transformed with 30 µg plasmid DNA and imaged by confocal microscopy 14–18 h after transformation in an Olympus FV1000 microscope (Olympus).

### Plant transformation

For complementation analyses, wild-type or mutant YFP–D6PK variants expressed from a *D6PK* promoter fragment (D6PKp) were transformed into *d6pk d6pkl1* by floral dip transformation^37^. Transgenic seedlings were selected using 1/2 Murashige and Skoog medium with 15 µg l^−1^ phosphinotricin. The resulting T1 transformants were propagated and analysed for phototropism response defects and cellular protein distribution using an Olympus FV1000 microscope with high sensitivity gallium arsenide phosphide detectors using identical acquisition settings between samples (Olympus). PDK1p::YFP–D6PK and PDK1p::YFP–D6PK_SMD were transformed into the progeny of a segregating *pdk1-14 pdk2-4/PDK2* line. Homozygous transgenic complementation lines in *pdk1-14 pdk2-4* were identified for further analysis by microscopy, as described above.

### Physiological experiments

Phototropic hypocotyl bending responses were examined in 3-day-old seedlings grown in the dark on 1/2 Murashige and Skoog medium. Etiolated seedling hypocotyls were straightened under safe-green light and then exposed to a unilateral light stimulus of 4.9 µmol m^−2^ s^−1^ in a FloraLED chamber (CLF Plant Climatics). The bending angle was calculated from scanned seedling images using the angle measurement tool of Fiji 2.9.0 (NIH ImageJ) and displayed on rose diagrams from the Origin 2020 software (OriginLab)^38^.

### Cell biology and quantification

To assess the quantitative distribution of YFP-tagged D6PK in the cell, the measure function of the NIH Image Fiji module was used, and grey values of selected regions of interest (ROIs) at the plasma membrane and intracellularly were measured and put in relation with each other^38^. Polarity indices were calculated with the Fiji profile plot function by establishing the ratio of the respective maxima from signal intensities measured through the basal and the lateral plasma membranes after subtraction of the average background. Profile blots were generated by laying a line through the cell, starting just below the apical (shootward) plasma membrane and passing through the basal plasma membrane. Measurements were performed in in five cells of three seedlings, considering the measurements from one seedling as biological replicate. The results from three biological replicate seedlings were sufficient for quantifications as determined by power analysis for 95% statistical power. To determine the effect of treatment or genotype, comparisons were made using a grouped analysis and one-way analysis of variance (ANOVA) to determine row and column effects. For co-localization measurements of YFP–D6PK and FM4-64, signals for selected ROIs were measured for the two channels in the cytosol and the plasma membrane in mock-treated and BFA-treated root epidermis cells using the Fiji ROI manager and measure function^39^. The measured signals were background-subtracted and the overlap calculated by subtracting the average signal from the measured signal in the ROIs for plasma membrane and intracellular regions individually and forming a ratio of normalized YFP and FM4-64 signal. The ratios were plotted to demonstrate the degree of co-localization in the cytosol, such that an increased ratio reflects an increase of YFP in the cytosol and a decrease reflects an increased signal at the plasma membrane. For co-localization determination, YFP (YFP–D6PK) and Cy3 (aS310p/S311p) signals of immunostained seedlings were determined using the Fiji JaCoP plug-in, by calculating the Pearson’s coefficient (*r*) and by generating profile plots of YFP and Cy3 signal in the same cell as described above^39^.

### FRAP

For FRAP, 5-day-old seedlings were analysed on a Leica SP8 (Leica Microsystems) using the FRAP function by bleaching a 4 µm × 1 µm window at the basal plasma membrane of root epidermal cells with a 514 nm Argon laser resulting in 80–90% bleach depth. The same acquisition settings between samples were used. Fluorescence recovery was quantified following 200 or 500 frames, with 0.233 s per frame, after photobleaching. Recovery was compared to five frames taken before bleaching. For image alignment and quantification, the 3D drift correction tool, the ROI manager and the measure tool of Fiji were used^38^. The resulting measurements were analysed with the easyFRAP Matlab package (GPL v3), using a full-scale normalization and double exponential fit for curve fitting^40^.

### Recombinant protein expression and purification

Recombinant GST–D6PK was obtained after transformation of *E. coli* strain Rosetta DE3 (Novagen) and, after the optical density of the LB24-grown culture had reached OD_600_ = 0.9, induction of protein expression with 0.5 mM iso-propyl-thio-galactoside for 18 h at 18 °C. Proteins were purified with an Äkta purifier (GE Healthcare) using a 1 ml GSTrap FF column (GE Healthcare) and eluted with 20 mM reduced glutathione (Sigma). After elution, the protein concentration was estimated by separating a fraction of the purified protein on a sodium dodecyl sulfate polyacrylamide gel electrophoresis (SDS–PAGE) gel using a dilution series prepared from albumin fraction V (Roth). For protein kinase reactions, 0.5 µg of purified recombinant GST–D6PK was used, alone or in combination with 0.5 µg GST–PIN1, as described previously^8^.

To measure in vitro D6PK phosphorylation, purified GST–D6PK was analysed by mass spectrometry without and with the addition of adenosine triphosphate (5 µM). As this analysis revealed background phosphorylation of recombinant GST–D6PK, purified GST–D6PK or a kinase-dead GST–D6PK_KE variant were treated with λ-phosphatase (200 units) for 20 min before incubation with recombinant GST–PDK1 and adenosine triphosphate (5 µM) to monitor for PDK1-dependent phosphorylation events. For the heat map, ratios of peptides found in phosphorylated and non-phosphorylated form in D6PK were calculated and normalized to the respective total peptide intensity. To estimate whether these are auto- or trans-phosphorylation sites, the ratio for D6PK was set to 1, and increases or decreases in relative phosphopeptide abundance in other variants were monitored compared to untreated GST–D6PK. The peptide corresponding to D6PK S310/S311 was recovered, due to the small size of the tryptic fragment, only very rarely and only in its phosphorylated form, rendering it impossible to establish the corresponding correlations.

### Auxin transport assays

Auxin transport assays in hypocotyls of etiolated seedlings were carried out as previously reported, with slight modifications^9^. Briefly, tritiated indole-3-acetic acid (IAA) ([3H]IAA; specific activity 25 Ci mmol^−1^; 1 mCi ml^−1^) (RC Tritec) was dissolved to a final concentration of 400 nM in 5 mM 2-(N-morpholino)ethanesulfonic acid (pH 5.5), 1% glycerol. Four-day-old dark-grown seedlings were placed onto 6 mm Parafilm (Beemis) strips placed on the surface of vertical plates containing 1/2 Murashige and Skoog medium. The seedlings were aligned on the parafilm strip so that the cotyledons and the apical part of the hypocotyl covered 5 mm of the parafilm strip, allowing for the application of 0.5 μl ^3^H–IAA solution to the cotyledons of each seedling. Seedlings were then incubated vertically in the dark for 4 h. Subsequently, the lower part of the hypocotyl was cut for scintillation counting. Five seedlings were grouped into one scintillation vial containing 2 ml Ultima Gold liquid scintillation cocktail and counted in a Tri-Carb 4910TR Liquid Scintillation counter (Perkin Elmer). For evaluation and representation of auxin transport data, the measured desintegrations per minute blank value was subtracted from the individual sample measurements. All measurements were normalized to the average determined for the wild type (Col-0), which was set to 100%.

### qRT-PCR

RNA for qRT-PCR was extracted using the NucleoSpin RNA Plant kit (Macherey–Nagel). About 1 µg RNA was reverse transcribed using an oligo(dT) primer and M-MuLV reverse transcriptase (Thermo Fisher Scientific). *D6PK* transcript levels were determined with the primers AG78 and AG79 from 3.125 ng cDNA equivalent using Takyon No ROX SYBR 2X MasterMix blue dTTP (Eurogentec) in a CFX384 Real-Time System Cycler (Bio-Rad). Amplification was performed using a four-step protocol (step 1, 50 °C for 3 min; step 2, 95 °C for 3 min; step 3, 95 °C for 15 sec; step 4, 60 °C for 40 sec) with 41 repeats of steps 3 and 4 and a final melt curve step from 65 °C to 95 °C for 5 sec at 0.5 °C increments. The results were normalized to *ACTIN2* and *ACTIN8* amplified using the primers D86 and D87. Results from three biological replicates are shown.

### Analysis of metal binding

For metal analysis, recombinant GST–GNC, GST–PIN1 HL^8^, GST–D6PK, GST–D6PK∆MID, GST–D6PK_MID^13^, GST–CPK21 and GST–CPK21 (short)^41^. were expressed and purified as described above. Proteins from three independent purifications were pooled, and three technical replicate measurements were performed with the pooled samples at a concentration of 1 mg ml^−1^ in 50 mM NaCl, 20 mM Tris HCl, pH 7.5. For element determination, the samples were gently thawed and diluted to 3 ml with milli-Q water. Measurements of the elements Ca^2+^ (spectral element line 183.801 nm), Fe^2+^ (259.941 nm), Mg^2+^ (279.079 nm) and Zn^2+^ (213.856 nm) were performed with inductively coupled plasma atomic emission spectrometry using an Optima 7300 DV (Perkin Elmer). Sample introduction was carried out with a peristaltic pump, connected to a micromist nebulizer with a cyclon spray chamber. The radio frequency power was set to 1,400 W, the plasma gas was 15 l Ar per min, and the nebulizer gas was 0.6 l Ar per min, optimized on a daily basis. For quality control, three blank determinations and a control determination of certified standards (CPI International) was performed after ten measurements for all elements (iron [Fe], order number 4095-1000262; magnesium [Mg], 4095-1000311; zinc [Zn], 4095-1000681; calcium [Ca], 4095-100091). Calculation of results was carried out on a computerized lab data management system, relating the sample measurements to calibration curves, blank determinations and control standards.

### Analysis of disulfide bond formation

Disulfide bond analysis was performed, as previously described with modifications^42^. Two reactions of 1 mg of total protein extract, extracted from 7-day-old seedlings expressing YFP–D6PK, were prepared in 150 mM NaCl, 0.5% Triton-X-100, 0.2% SDS, 1 mM phenylmethylsulfonyl fluoride, 1 mM ethylenediamine tetraacetic acid (EDTA), 1× Protease Inhibitor Cocktail and 50 mM 4-2-hydroxyethyl-1-piperazineethanesulfonic acid, pH 7.5 (Sigma). One reaction included 20 mM TCEP (Tris-[2-carboxyethyl]-phosphin; reduced) to reduce disulfides that may be present in the purified protein before subjecting the extract to the procedures described above. Free cysteines were blocked using 100 mM NEM (N-ethylmaleimide) for 4 h before stopping the reaction by incubating with 20 mM 1,4 dithiothreitol for 30 min. The protein was diluted fivefold and immunoprecipitated using green fluorescent protein (GFP)-Trap magnetic agarose (Chromotek). After mass spectrometry, the comparative analysis between the NEM-treated only and the NEM-plus-TCEP-treated samples provided a quantitative readout for the presence of disulfide bonds and for the relative efficiency of NEM labelling of individual cysteines^42,43^.

### Analysis of S-acylation

The acyl biotin exchange assay was performed as previously described using 1 mg of total protein obtained from transgenic lines overexpressing YFP–D6PK^44^. Protein extracts were treated with NEM to modify free cysteines, which was followed by hydroxylamine treatment leading to the hydrolysation of S-acylations. Subsequently, the extract was labelled with sulfhydryl-reactive biotin–HPDP ((3aS,4S,6aR)-hexahydro-2-oxo-N-[6-[[1-oxo-3-(2-pyridinyldithio)propyl]amino]hexyl]-1H-thieno[3,4-d]imidazole-4-pentanamide), which reacts with the thiol groups of unmodified cysteines; the modified proteins were purified with streptavidin beads and subjected to anti-GFP immunoblot analysis. For the negative control, the hydroxylamine treatment was omitted; tubulin A (TUA), a known palmitoylation substrate detected with aTUA (anti-tubulin alpha chain, AS10680, Agrisera), served as a positive control^27^. To assess dynamic S-acylation of YFP–D6PK, an acyl resin-assisted capture assay was performed using soluble and membrane fractions obtained after ultracentrifugation of 1 mg total at 100,000 *g* protein as previously described^45^. 2-Bromo palmitate (2-BP) treatments were performed by treating 6-day-old light-grown seedlings for 2 h with 2-BP, as specified and diluted from a 100 mM stock solution prepared in ethanol (Sigma), before examining the seedlings with an Olympus FV1000 confocal laser scanning microscope (Olympus). Protein fractionation, immunoblot and quantification of 2-BP-treated samples were performed as described above.

### Immunoblot analysis and immunostaining

For immunoblot analysis of plant protein extracts, YFP–D6PK or its variants were examined by SDS–PAGE using 40 µg total protein and, where specified, after subcellular fractionation through 1 h ultracentrifugation at 100,000 *g*. SDS–PAGE were blotted and probed with anti-GFP (aGFP; 1:3,000, laboratory stock), aTUA (AS10 680; 1:2,000, Agrisera) or anti-uridine diphosphate (UDP) glucose pyrophosphorylase (aUGP, AS05 086; 1:1500, Agrisera) primary antibodies and an anti-rabbit horse radish peroxidase-conjugated secondary antibody (1:100,000; A9169; Sigma). Chemiluminescence was detected with a Fujifilm LAS 4000 mini (Fuji) and quantified using the measure and profile functions of the Fiji (ImageJ) software^38^.

To assess D6PK phosphorylation at S310/S311, an anti-aS310p/S311p antibody was raised in rabbits against the respective phosphorylated chemically synthesized peptide (H-CPRFF-phosphoS-phosphoS-KSKKDK-NH_2_) and validated by enzyme-linked immunosorbent assay (Eurogentec).

For immunoblot analysis of recombinant protein, GST–D6PK, GST–D6PK_SSAA and GST–D6PK_SSDD were purified as described previously. For λ-phosphatase treatments, GST–D6PK was treated with 400 U λ-phosphatase (New England Biolabs) for 20 min at room temperature with the protein bound to glutathione agarose before washing and elution. Equal protein amounts were loaded on an SDS–PAGE and blotted and probed with anti-aS310p/S311p (1:250) or anti-glutathione S-transferase (aGST, 27-4577-01; 1:2,000, Cytiva) primary antibodies and anti-rabbit horse radish peroxidase-conjugated (1:100,000; A9169; Sigma) or anti-rabbit alkaline phosphatase-conjugated secondary antibodies (1:1,000, A3937; Sigma). Immunoblots were imaged using a Fujifilm LAS 4000 mini (Fuji).

### Immunohistochemistry

For immunostaining, 5-day-old light-grown seedlings were treated with 10 µM BFA or a corresponding mock solution for 30 min and fixed for 1 h at room temperature under vacuum in 4% (*v*/*v*) paraformaldehyde in PBS pH 7.4. Cell walls were partially digested for 30 min at 37 °C with 2% (*w*/*v*) Driselase (Sigma). Plasma membranes were permeabilized for 1 h at room temperature with 3% (*v*/*v*) Nonidet P-40 (AppliChem) in 10% (*v*/*v*) DMSO/PBS. The samples were blocked for 1 h at room temperature with 4% (*w*/*v*) BSA in PBS before anti-aS310p/S311p (1:100), diluted in blocking solution, was added for 4 h at 37 °C. Following washes with 0.1% Triton X-100 (AppliChem) in PBS, 4 h incubation at 37 °C with a Cy3-conjugated anti-rabbit antibody (1:600; Dianova) was done for primary antibody detection. Following renewed washes, the immunostained seedling roots were examined in an Olympus FV1000 confocal laser scanning microscope (Olympus). For the quantification of co-localization, YFP (YFP–D6PK) and Cy3 (aS310p/S311p) signals of immunostained seedlings were determined using the Fiji JaCoP plug-in and by calculating the Pearson’s correlation coefficient (*r*).

### Mass spectrometry

For phosphoproteomics analysis of in vitro phosphorylated GST–D6PK, in-gel trypsin digestion was performed according to standard procedures^46^. Briefly, the samples were run on a NuPAGE 4–12% Bis–Tris protein gel (ThermoFisher Scientific) for 5 min. Subsequently, the proteins were separated on a short SDS–PAGE gel and, for identification of phosphosites, separated on a long SDS–PAGE gel. The protein gel slice was excised and reduced with 50 mM 1,4 dithiothreitol, alkylated with 55 mm chloroacetamide and digested overnight with trypsin. The tryptic peptides were eluted and dried in a vacuum concentrator and dissolved in 0.1% (*v*/*v*) formic acid in high-performance liquid chromatography (HPLC)-grade water before liquid chromatography–mass spectrometry analysis.

Liquid chromatography-tandem mass spectroscopy analysis was performed on Orbitrap mass spectrometer systems (Thermo Fisher Scientific) coupled on-line to a Dionex 3000 HPLC (Thermo Fisher Scientific) with a 75 μm × 2 cm trap column (Reprosil Pur ODS-3 5 μm particles (Dr. Maisch HPLC)) and a 75 μm × 40 cm analytical column (3 μm particles C18 Reprosil Gold 120 (Dr. Maisch HPLC)). Peptides were separated at a flow rate of 300 nl min^−1^ over a 50 min gradient from 4% to 32% acetonitrile in 5% dimethylsulfoxide, 0.1% formic acid, followed by a washing step (column temperature 50 °C). For the in vitro D6PK redox and kinase assay samples, full-scan mass spectra (*m*/*z* 360–1,300) were acquired in profile mode on an Orbitrap Fusion Lumos Tribrid Mass Spectrometer with 60,000 resolution, an automatic gain control target value of 5 × 10^5^ or 4 × 10^5^ and 10 or 50 ms maximum injection time, respectively. For the top 20 precursor ions, Orbitrap readout MS2 scans were performed, using higher-energy collisional dissociation (HCD) fragmentation with 28% or 30% normalized collision energy, 15,000 resolution, an automatic gain control target value of 2 × 10^5^ or 5 × 10^4^, 1.7 or 1.3 *m/z* isolation width and 50 ms, 10 ms or 22 ms maximum injection time, respectively. The minimum intensity threshold was set to 2 × 10^4^ with a dynamic exclusion of 20 s. For the EDTA kinase assay samples, the dynamic exclusion was set to 10 ms for precursors from a customized inclusion mass list and 60 ms for all other precursors. Immunoprecipitates from the D6PK redox assay were measured with comparable settings on a Q Exactive HF (ThermoFisher). Here the MS1 automatic gain control target value was set to 3 × 10^6^, the normalized collision energy was 25% and MS2 spectra were acquired at 30,000 resolution. To confirm and more precisely monitor phospho-modified peptides identified in the data-dependent acquisition analysis of the EDTA kinase assay samples, a targeted Parallel Reaction Monitoring (PRM) was set up on the Orbitrap Fusion Lumos Tribrid Mass Spectrometer (Thermo Fisher) using the same liquid chromatography gradient settings as described above. PRM measurements were performed with the acquisition method switching between experiments after one duty cycle. The first experiment consisted of a full-scan MS1 spectrum recorded in the orbitrap (360 to 1,300 *m*/*z*, 15,000 resolution, automatic gain control target value of 4 × 10^5^, maximum injection time 10 ms), followed by a targeted MS2 PRM scan triggering MS2 scans based on a list containing retention time window, *m*/*z* and charge information from the previous data-dependent acquisition experiment. For the targeted mass spectrometry analysis 2 (tMS2) PRM scan, the scheduled precursors were isolated (isolation window 0.7 *m*/*z*), fragmented via HCD (normalized collision energy [NCE] 28%) and recorded in the Lumos orbitrap (120 to 2,000 *m*/*z*, 15,000 resolution, automatic gain control target 2 × 10^5^, maximum injection time 100 ms).

Peptide and protein identification and quantification were performed with MaxQuant^47^ using standard settings (version 1.5.8.3 in the case of the redox assay, version 1.6.3.3 in the case of the kinase assays). Raw files were searched against the Araport11 database (Araport11_genes.201606.pep.fasta) at www.arabidopsis.org and common contaminants. An *E. coli* reference database (562_Escherichiacoli_NCBI. fasta) was added when recombinantly expressed proteins were analysed. The D6PK–PDK1 kinase experiment was searched against a custom database with the protein sequences of D6PK, PIP5K1 and PDK1. Cysteine modification with carbamidomethyl (+57.0214) or N-ethylmaleimide (+125.0476) as well as oxidation of methionine and N-terminal protein acetylation were set as variable modifications for the redox experiments. For the in vitro kinase assay samples, carbamidomethylation of cysteine was set as fixed, and phosphorylation of serine, threonine or tyrosine as variable modification. Trypsin/P was specified as the proteolytic enzyme, with up to two missed cleavage sites allowed. The match between run function was enabled. Results were filtered to 1% peptide spectra matched, protein and site false discovery rate. Mass spectra were displayed with MaxQuant viewer^48^. The mass spectrometry proteomics data have been deposited to the ProteomeXchange Consortium via the PRIDE partner repository with the dataset identifier PXD037885 (ref. ^49^). RAW files from the PRM measurement were imported into Skyline (64 bit)^50^ for data filtering and analysis. Peaks were integrated using the automatic peak-finding function followed by manual curation of all peak boundaries and transitions. The summed area under the fragment ion traces was exported for data visualization in Microsoft Excel (v. 16.51).

### Reporting summary

Further information on research design is available in the Nature Portfolio Reporting Summary linked to this article.

### Supplementary information

Supplementary Information Supplementary Figs. 1–3, legends and Supplementary Table 1.

Reporting Summary

### Source data

Source Data Fig. 3 Unprocessed western blots.

Source Data Fig. 3 Unprocessed western blots.

Source Data Extended Data Fig. 4 Unprocessed western blots.

## Data Availability

All material, data, accession codes, unique identifiers or web links for publicly available datasets will be made available upon request, unless not already provided in the article. The mass spectrometry proteomics data have been deposited to the ProteomeXchange Consortium via the PRIDE partner repository with the dataset identifier PXD037885. Source data are provided with this paper.
