# Supplementary material for: D6PK plasma membrane polarity requires a repeated CXX(X)P motif and PDK1-dependent phosphorylation
Source: Nat Plants. 2024 Jan 26;10(2):300–14. doi: 10.1038/s41477-023-01615-6 (PMC10881395; doi:10.1038/s41477-023-01615-6)
Supplement: Supplementary file 1 — Supplementary Figs. 1–3, legends and Supplementary Table 1. [file 41477_2023_1615_MOESM1_ESM.pdf]

nature plants

Article https://doi.org/10.1038/s41477-023-01615-6

D6PK plasma membrane polarity requires a repeated CXX(X)P motif and PDK1-dependent phosphorylation

In the format provided by the authors and unedited

Supplementary Figure 1

A

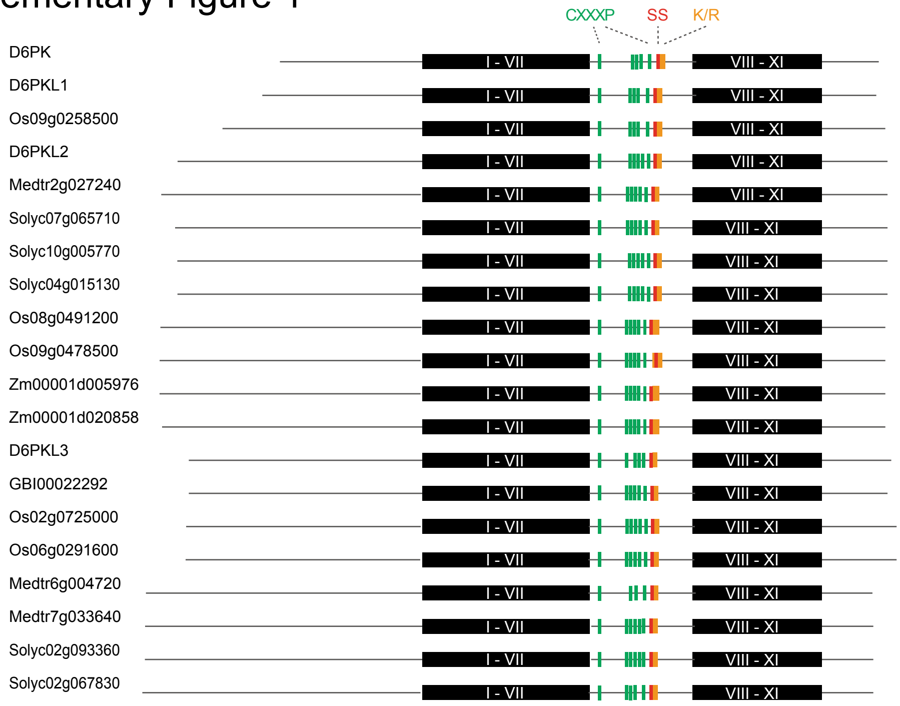

B

```
                   CXXXP                                   CXXXP  CXXXP  CXXXP        CXXP           SS   K/R
               DFD C1     P1                               C2    P2C3   P3C4    P4   C5 P5                                          SMS
D6PK           DFDLSLRCTVSLSIVRSANVGSEGLSKNS-VSCSQQPACIQQPSCISMAP-----TSCFGPRFF------SSKSKKDKKPKTENGNHQVTPLPELVAEPTGARSMS
D6PKL1         DFDLSLRCTVSPTVVRSTVLASEG-QKNS-GYCA-QPACIQQPSC-ISAP-----TTCFSPRYF------SSKSKKDKKMKNETGN-QVSPLPELVAEPTSARSMS
Os09g0258500   DFDLSLRCAVSPTLLKSSNPGVDPNQKGNPSYCV-QPVCIE-PAC-IQ-PSCVTTTTCFAPRFF------SSKSKKEKKAKTDIAS-QVRPLPELVAEPTDARSMS
D6PKL2         DFDLSLRCLVSPTLVKSAAIESDPLRKN--VYCV-QPACIE-PSC-IQ-PSCTVPTTCFSPRLF------SSKSKKDRKPKNDTAN-QVRPLPELVAEPTDARSMS
Medtr2g027240  DFDLSLRCAVSPTLVKSSSIDSEPLRKNT-GYCA-QPACIEPPSC-IQ-PSCVAPTSCFSPRLF------SSKSKKERKAKTELGN-QVSPLPELIAEPTDARSMS
Solyc07g065710 DFDLSLRCAVSPTLVKSSSLEGEPLRKNS-AYCV-QPACIE-PSC-IQ-PSCAVTTTCFGPRLF------SSKSKKEKKSKNEIGN-QVSPLPELIAEPTGARSMS
Solyc10g005770 DFDLSLRCAVSPTLVKLSSIDVEPLRKNS-GYCV-QPTCIE-PSC-IQ-PSCAVTTSCFGPRFF------SSKSKKEKKSKNDTGN-QVSPLPELMAEPTGARSMS
Solyc04g015130 DFDLSLCCSFSPTLVKTSSLELEPLKKNS-GYCV-QPACIE-PSC-IQ-PSCVVPTTCFSRRIF------SSKSKKDRKPKNDVGN-QVRPLPELMAEPTSARSMS
Os08g0491200   DFDLSLRCAVSPTVVKSANPGPDALQRNNQAYCV-QPACIQ-PSC-IQ-PSCVAPTTCFGPRFF--SSKSKSKSKKEKKSKPEVVN-QISPLPELIAEPTDARSMS
Os09g0478500   DFDLSLRCSVSPTVIKSANPGLDALQRNNAAYCV-QPACIE-PSC-IQ-PSCVAPTTCFGPRFF---KSKSKSKSKKEKSKPEAAN-QASLFPELIAEPTDARSMS
Zm00001d005976 DFDLSLRCSVSPTVIKSANPGLDAMQRNNAAYCA-QPACIE-PSC-IQ-PSCVAPTTCFGPRFFSKSKSKSKSKSKKDKSKPDAPN-QENLFPELIAEPTDARSMS
Zm00001d020858 DFDLSLRCSVSLTVIKSANPGLDALQRNNAAYCA-QPACIE-PSC-IQ-PSCVAPTTCFGPRFFSKSKSKSKSKPKKEKSKPDGPN-QENLFPELIAEPTDARSMS
D6PKL3         DFDLSLRCAVSPTLVRFAAITLES--KSS-SYCI-QPTCVDQSSCIVQ-PDCIQP-VCFTPRFL--------SKGKHRKKSNDMSR-QIRPLPELIAEPTSARSMS
GBI00022292    DFDLSLRCVVSPTLVKSASSSVDPSRRAP-VYCV-QPSCIE-PVC-VR-PPCIQP-SCFVPRFL------PQRSKKTRKPKNEIVN-QATPLPELIAEPTSARSMS
Os02g0725000   DFDLSLRCAVSPTLIKSSNPDAEALRKNSQGYCV-QPACVE-PSCVIQ-PSCAAPTTCFGPRFF-------SKSKKDRKPKPEIAT-QISPWPELIAEPSDARSMS
Os06g0291600   DFDLSLRCAVSPTLIRSSNPDAEALRKNNQAYCV-QPACVE-PSCMIQ-PSCATPTTCFGPRFF-------SKSKKDRKPKPEVVN-QVSPWPELIAEPSDARSMS
Medtr6g004720  DFDLSLRCTVSPTLVKSSNPITET--KSS-GY------CIQ-PACAMQ-PDCIQH-ACFSPRFL-----SGKSKEKKFRLKNDMHH-QMTPLPELVAEPTSARSMS
Medtr7g033640  DFDLSLRCAVSPTLVKSANSNLET--KGS-GYCA-QPACIE-PTCVIK-PDCIQP-SCFTPRFL---SGKSKKKEKKLKPKNDVHN-QVTPLPELMAEPTNARSMS
Solyc02g093360 DFDLSLRCAVSPTLVRSSNSSLEC--KSS-SYCV-QPACIE-PSCVVQ-PACIQP-SCFTPRFL---S-KNKKEKKS-KQKTETYNQVNRPLPELLAEPTSARSMS
Solyc02g067830 DFDLSLRCAVSPTLVKSSNSSLES--KTS-SYCV-QPACIE-PSCVIQ-P------ACFSPRFL-----RPKKGKKT-KQKSEMHNQVS-PLPELMAEPTNARSMS
```

C

```
                   CXXXP                                                CXXXP         SS   K/R
               DFD C1     P1                                            C4    P4    P5                                     SMS
AGC1.5         DFDLSLRCTFNPTLVKSSSVCSGGGA--------ILNEEFAV---NGCMHPSA-FLPRLL-PSKKTRKAKSDSGL--GGL----------SMPELMAEPTDVRSMS
Os05g14750     DFDLSLRCTVCPTLVKSSSVHATGSGGGIGSRGDAIDGGESMPANQGCIQPSS-FFPRIL-PRRSRKASKSDMGLLLNGAA----------AVEFNAEPTEARSMS
Os02g18430     DFDLSLRCSVSPALVRSPSGRVGAGAGLV----------------HGCVLPR--ILPRRSGKKKKKQKGNDQEVTSATGDGNGKNRPPPATSLEFTAEPTGARSMS
Os01g13270     DFDLSLRCSVCPMLVKSSSVHAGANGVVKGLAAGGGGDGEGV--GVGCMQPSA-FLPRIL-PKRSRKTSKSDLGLLHG-S-----------PLEFNAEPTDARSMS
AGC1.6         DFDLSLRCSVNPTLVKSFN---GGGTT------GIIDDNAAV---QGCYQPSA-FFPRMLQSSKKNRKSKSDFD-----G-----------SLPELMAEPTNVKSMS
AGC1.7         DFDLSLRCSVNPTLVKSSSVHGGGGGGNPSSGSGILDNDNAV---QGCIQPST-FFPRNILPTKKNRKSKSDFGLFVGGS-----------LPELMAEPTNVRSMS
Medtr8g446990  DFDLSLRCSVSPTLVKSSSAHITTTTTTTSIA--ILDDEHAV---QGCMQPSNFFFPRIL-PYKRNRKSKSDFGLMVGGR-----------LPELMAEPTNVRSMS
Medtr2g062850  DFDLSLRCSVCPTLVKSSSTH-GGNSSGNSDSGGILNDDQAV-----IAQSSTSFFPRIL-PSKKNRKAKSDFGLLVNGN-----------RLPELMAEPTNVRSMS
Solyc12g017390 DFDLSLRCSVSPTLVKSSSVHAAGGGSGSSRPVGLIDEDAAV---QGCIQPST-FFPRILQSSKKNRKAKSDFGLFVNGS-----------MPELMAEPTNVKSMS
```

Supplementary Figure 2

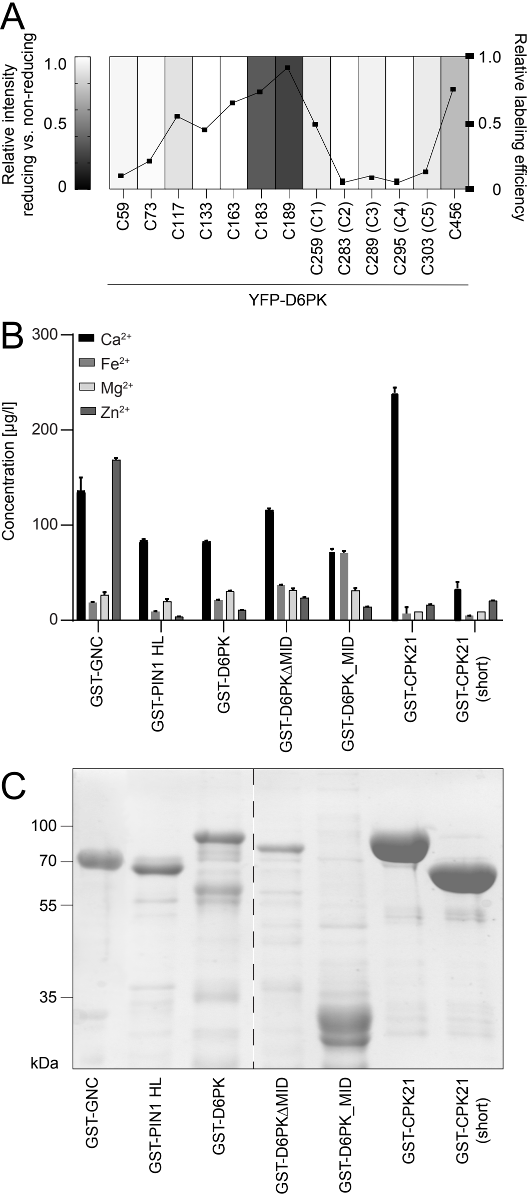

Supplementary Figure 3

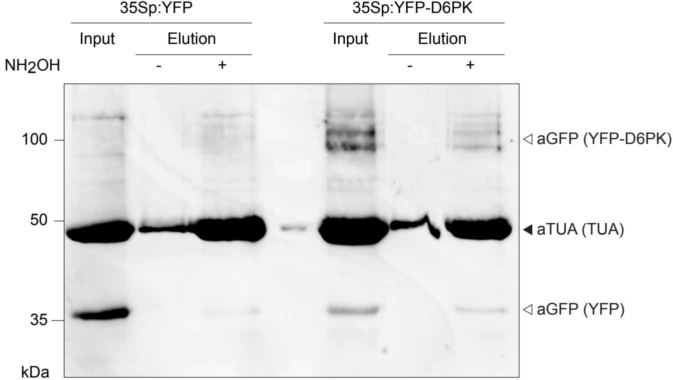

SUPLLEMENTARY FIGURE LEGENDS

Supplementary Figure 1. The numbers and positions of CXX(X)P motif repeats are conserved among AGC1 kinase subfamilies from vascular plants. A and **B.** Schematic representation of putative D6PK orthologous kinases from rice (*Oryza sativa*, Os), *Medicago truncatula* (*Medtr*), tomato (*Solanum lycopersicon*, Solyc), gingko (*Gingko biloba*, GB) (A) and their middle domain Muscle sequence alignment (B). **C.** Middle domain sequence alignment of rice, tomato and Medicago orthologous kinases of *Arabidopsis thaliana* AGC1.5 – AGC1.7. CXX(X)P motifs, as well as adjacent serine residues and polybasic motifs (K/R) are highlighted. The conserved protein kinase subdomains I – VII and VIII – XI are marked by boxes (A) and key residues of subdomains VII (DFD) and VIII (SMS) are included in the alignments (B and C).

Supplementary Figure 2. The D6PK middle domain does not engage in disulfide bond formation or cation complexation. A. Result from a mass spectrometry-based analysis for disulfide bond formation showing reducing to non-reducing properties of the individual cysteines as specified (grey scale and grey bars) together with their relative labelling efficiency. Note that CXX(X)P motif cysteines C1 – C5 are not efficiently labelled. **B.** Graph displaying the average and standard deviation of ionometric analyses with recombinant purified GST-tagged proteins as specified. Note that there is no qualitative difference in cation binding between GST-D6PK and GST-D6PKΔMID. Data are derived from three technical replicates obtained from three pooled purifications. **C.** Image of a Coomassie Brilliant Blue-stained gel with purified recombinant proteins, as specified, and used in the ionometric study as shown in (B). GST-GNC (GATA, NITRATE-INDUCIBLE, CARBON METABOLISM-INVOLVED) is a GST-fusion of a Zn^{2+}-finger transcription factor[51]; GST-PIN1 HL is a GST-fusion with the hydrophilic loop of PIN1 (PIN-FORMED1) and serves as a negative control; GST-D6PKΔMID is the middle domain deletion variant of GST-D6PK; GST-D6PK_MID is a GST-fusion of the D6PK middle domain; GST-CPK21 corresponds to a GST-fusion of a Ca^{2+}-binding protein kinase[41]; GST-CPK21 (short) represents a truncated version of GST-CPK21 lacking the Ca^{2+}-binding domain. The dotted line marks splicing of the gel to remove irrelevant sample lanes.

Supplementary Figure 3. Control experiment with 35Sp:YFP and 35Sp:YFP-D6PK reveals only marginal binding of YFP in an acetyl biotin exchange assay. Immunoblot of total protein inputs and protein elutions after an acetyl biotin switch (of protein extracts prepared from transgenic plants expressing YFP or YFP-D6PK as specified. Anti-GREEN FLUORESCENT PROTEIN (aGFP) recognizes the YFP-tag; anti-tubulin A (aTUA) recognizes the soluble palmitoylated tubulin A protein. The experiment was repeated twice with similar outcome.

Supplementary Table 1. List of primers used in this study.

Primer Name	Primer Sequence
D6PK C1S mutagenesis	[Phos]TCCCTGAGATCTACTGTGAGC
D6PK C2S mutagenesis	[Phos]TCGGTTTCTTCTTCGCAACAACC
D6PK C3S mutagenesis	[Phos]ACAACCTGCATCTATCCAGCAAC
D6PK C4S mutagenesis	[Phos]AGCAACCATCTTCTATCTCAATGGC
D6PK C5S mutagenesis	[Phos]CTCCAACATCTTCTTCGGTCCTCG
D6PK C1A mutagenesis	[Phos]TCCCTGAGAGCTACTGTGAGC
D6PK C5A mutagenesis	[Phos]TGATGGAGTTTGCTCCTGGTG
D6PK P2G mutagenesis	[Phos]GCTCGCAACAAGGTGCATGCATC
D6PK P3G mutagenesis	[Phos]ATCCAGCAAGGATCTTGCATCTC
D6PK P4G mutagenesis	[Phos]CAATGGGCTGGAACATCTTGCTTCG
D6PK P5G mutagenesis	[Phos]TTGCTTCGGTGGTCGGTTCTTC
D6PK SSAA mutagenesis	[Phos]GGTCCTCGGTTCTTCGCAGCGAAATCCAAGAAAGACA
D6PK SSDD mutagenesis	[Phos]GGTCCTCGGTTCTTCGACGATAAATCCAAGAAAGAC
GST-GNC cloning forward	TAAGCAGAATTCATGGATTCAAATTTTCATTAC
GST-GNC cloning reverse	TGCTTAGTCGACTCAACCGTGAACCATTCCATA
D6PKΔSAN deletion reverse	GACCGAAGCAAGATGTTCTGACTATAGAGAGGCTCACAG
D6PKΔSAN deletion forward	CTGAGCATTGTTCGTACCAGCTGTTTTGGTCCGCGT
D6PK-attB1 Gateway primer	GGGGACAAGTTTGTACAAAAAAGCAGGCTTCATGATGGCTTCAAAAACTCC
D6PK-attB2 Gateway primer	GGGGACCACTTTGTACAAGAAAGCTGGGTTTCAGAAGAAATCAAACTCAAG
D6PK_SMD forward	GGCGCCCGTTCTATGGATTTTGTTGGCACACAC
D6PK_SMD reverse	GTGTGTGCCAACAAAATCCATAGAACGGGCGCC
D6PK qRT forward	TTGCTTGTGAAGGAGCCACA
D6PK qRT reverse	TGCAGTGTCTTGATCACC
ACTIN2 – 8 q RT forward	GGTAACATTGTGCTCAGTGGTGG
ACTIN2 – 8 q RT reverse	AACGACCTTAATCTTCATGCTGC
