# Supplementary material for: D6PK plasma membrane polarity requires a repeated CXX(X)P motif and PDK1-dependent phosphorylation
Source: Nat Plants. 2024 Jan 26;10(2):300–14. doi: 10.1038/s41477-023-01615-6 (PMC10881395; doi:10.1038/s41477-023-01615-6)
Supplement: Supplementary file 2 — Reporting Summary [file 41477_2023_1615_MOESM2_ESM.pdf]

nature portfolio

Corresponding author(s): Claus Schwechheimer

Last updated by author(s): Claus Schwechheimer on Dec 05, 2023

Reporting Summary

Nature Portfolio wishes to improve the reproducibility of the work that we publish. This form provides structure for consistency and transparency in reporting. For further information on Nature Portfolio policies, see our Editorial Policies and the Editorial Policy Checklist.

Statistics

For all statistical analyses, confirm that the following items are present in the figure legend, table legend, main text, or Methods section.

n/a	Confirmed	
☐	☒	The exact sample size (*n*) for each experimental group/condition, given as a discrete number and unit of measurement
☐	☒	A statement on whether measurements were taken from distinct samples or whether the same sample was measured repeatedly
☐	☒	The statistical test(s) used AND whether they are one- or two-sided<br>*Only common tests should be described solely by name; describe more complex techniques in the Methods section.*
☒	☐	A description of all covariates tested
☐	☒	A description of any assumptions or corrections, such as tests of normality and adjustment for multiple comparisons
☐	☒	A full description of the statistical parameters including central tendency (e.g. means) or other basic estimates (e.g. regression coefficient) AND variation (e.g. standard deviation) or associated estimates of uncertainty (e.g. confidence intervals)
☐	☒	For null hypothesis testing, the test statistic (e.g. *F*, *t*, *r*) with confidence intervals, effect sizes, degrees of freedom and *P* value noted<br>*Give P values as exact values whenever suitable.*
☒	☐	For Bayesian analysis, information on the choice of priors and Markov chain Monte Carlo settings
☒	☐	For hierarchical and complex designs, identification of the appropriate level for tests and full reporting of outcomes
☐	☒	Estimates of effect sizes (e.g. Cohen's *d*, Pearson's *r*), indicating how they were calculated

Our web collection on statistics for biologists contains articles on many of the points above.

Software and code

Policy information about availability of computer code

Data collection	Fiji (NIH ImageJ) (Ref. 38), PLAZA4.0 (Ref. 33), Fujifilm LAS 4000 mini (Fuji, Japan)
Data analysis	Origin software (OriginLab Corporation, Northampton, MA, USA), Geneious R11.1.3 , easyFRAP Matlab (Ref. 40), Skyline (64-bit) (Ref. 50)

For manuscripts utilizing custom algorithms or software that are central to the research but not yet described in published literature, software must be made available to editors and reviewers. We strongly encourage code deposition in a community repository (e.g. GitHub). See the Nature Portfolio guidelines for submitting code & software for further information.

Data

Policy information about availability of data

All manuscripts must include a data availability statement. This statement should provide the following information, where applicable:

- Accession codes, unique identifiers, or web links for publicly available datasets
- A description of any restrictions on data availability
- For clinical datasets or third party data, please ensure that the statement adheres to our policy

DATA AVAILABILITY: All material, data, accession codes, unique identifiers, or web links for publicly available datasets will be made available upon request, unless not already provided in the article. The mass spectrometry proteomics data have been deposited to the ProteomeXchange Consortium via the PRIDE partner repository with the dataset identifier PXD037885.

Research involving human participants, their data, or biological material

Policy information about studies with human participants or human data. See also policy information about sex, gender (identity/presentation), and sexual orientation and race, ethnicity and racism.

Reporting on sex and gender	Does not apply
Reporting on race, ethnicity, or other socially relevant groupings	Does not apply
Population characteristics	Does not apply
Recruitment	Does not apply
Ethics oversight	Does not apply

Note that full information on the approval of the study protocol must also be provided in the manuscript.

Field-specific reporting

Please select the one below that is the best fit for your research. If you are not sure, read the appropriate sections before making your selection.

☒ Life sciences ☐ Behavioural & social sciences ☐ Ecological, evolutionary & environmental sciences

For a reference copy of the document with all sections, see nature.com/documents/nr-reporting-summary-flat.pdf

Life sciences study design

All studies must disclose on these points even when the disclosure is negative.

Sample size	To determine sample size, power analysis was performed, aiming at 95% statistical power
Data exclusions	No data were excluded.
Replication	The number of independent experiment replications is stated in the legend. In each case, comparable or similar outcome was obtained.
Randomization	Randomization was not applicable because plants were grown in uniform growth conditions, often bulked for extractions or using sufficiently large sample sizes.
Blinding	Randomization was not applicable because plants were grown in uniform growth conditions, often bulked for extractions or using sufficiently large sample sizes.

Behavioural & social sciences study design

All studies must disclose on these points even when the disclosure is negative.

Study description	*Briefly describe the study type including whether data are quantitative, qualitative, or mixed-methods (e.g. qualitative cross-sectional, quantitative experimental, mixed-methods case study).*
Research sample	*State the research sample (e.g. Harvard university undergraduates, villagers in rural India) and provide relevant demographic information (e.g. age, sex) and indicate whether the sample is representative. Provide a rationale for the study sample chosen. For studies involving existing datasets, please describe the dataset and source.*
Sampling strategy	*Describe the sampling procedure (e.g. random, snowball, stratified, convenience). Describe the statistical methods that were used to predetermine sample size OR if no sample-size calculation was performed, describe how sample sizes were chosen and provide a rationale for why these sample sizes are sufficient. For qualitative data, please indicate whether data saturation was considered, and what criteria were used to decide that no further sampling was needed.*
Data collection	*Provide details about the data collection procedure, including the instruments or devices used to record the data (e.g. pen and paper, computer, eye tracker, video or audio equipment) whether anyone was present besides the participant(s) and the researcher, and whether the researcher was blind to experimental condition and/or the study hypothesis during data collection.*
Timing	*Indicate the start and stop dates of data collection. If there is a gap between collection periods, state the dates for each sample cohort.*
Data exclusions	*If no data were excluded from the analyses, state so OR if data were excluded, provide the exact number of exclusions and the*

Data exclusions	rationale behind them, indicating whether exclusion criteria were pre-established.
Non-participation	State how many participants dropped out/declined participation and the reason(s) given OR provide response rate OR state that no participants dropped out/declined participation.
Randomization	If participants were not allocated into experimental groups, state so OR describe how participants were allocated to groups, and if allocation was not random, describe how covariates were controlled.

Ecological, evolutionary & environmental sciences study design

All studies must disclose on these points even when the disclosure is negative.

Study description	Briefly describe the study. For quantitative data include treatment factors and interactions, design structure (e.g. factorial, nested, hierarchical), nature and number of experimental units and replicates.
Research sample	Describe the research sample (e.g. a group of tagged Passer domesticus, all Stenocereus thurberi within Organ Pipe Cactus National Monument), and provide a rationale for the sample choice. When relevant, describe the organism taxa, source, sex, age range and any manipulations. State what population the sample is meant to represent when applicable. For studies involving existing datasets, describe the data and its source.
Sampling strategy	Note the sampling procedure. Describe the statistical methods that were used to predetermine sample size OR if no sample-size calculation was performed, describe how sample sizes were chosen and provide a rationale for why these sample sizes are sufficient.
Data collection	Describe the data collection procedure, including who recorded the data and how.
Timing and spatial scale	Indicate the start and stop dates of data collection, noting the frequency and periodicity of sampling and providing a rationale for these choices. If there is a gap between collection periods, state the dates for each sample cohort. Specify the spatial scale from which the data are taken
Data exclusions	If no data were excluded from the analyses, state so OR if data were excluded, describe the exclusions and the rationale behind them, indicating whether exclusion criteria were pre-established.
Reproducibility	Describe the measures taken to verify the reproducibility of experimental findings. For each experiment, note whether any attempts to repeat the experiment failed OR state that all attempts to repeat the experiment were successful.
Randomization	Describe how samples/organisms/participants were allocated into groups. If allocation was not random, describe how covariates were controlled. If this is not relevant to your study, explain why.
Blinding	Describe the extent of blinding used during data acquisition and analysis. If blinding was not possible, describe why OR explain why blinding was not relevant to your study.

Did the study involve field work? ☐ Yes ☐ No

Field work, collection and transport

Field conditions	Describe the study conditions for field work, providing relevant parameters (e.g. temperature, rainfall).
Location	State the location of the sampling or experiment, providing relevant parameters (e.g. latitude and longitude, elevation, water depth).
Access & import/export	Describe the efforts you have made to access habitats and to collect and import/export your samples in a responsible manner and in compliance with local, national and international laws, noting any permits that were obtained (give the name of the issuing authority, the date of issue, and any identifying information).
Disturbance	Describe any disturbance caused by the study and how it was minimized.

Reporting for specific materials, systems and methods

We require information from authors about some types of materials, experimental systems and methods used in many studies. Here, indicate whether each material, system or method listed is relevant to your study. If you are not sure if a list item applies to your research, read the appropriate section before selecting a response.

Materials & experimental systems

n/a	Involved in the study	
☐	☒	Antibodies
☒	☐	Eukaryotic cell lines
☒	☐	Palaeontology and archaeology
☒	☐	Animals and other organisms
☒	☐	Clinical data
☒	☐	Dual use research of concern
☐	☒	Plants

Methods

n/a	Involved in the study	
☒	☐	ChIP-seq
☒	☐	Flow cytometry
☒	☐	MRI-based neuroimaging

Antibodies

Antibodies used

anti-S310p/S311p (this study; Eurogentec, Liege, Belgium); anti-GFP (aGFP; dilution 1:3000, laboratory stock); anti-Tubulin A (aTUA, AS10 680; dilution 1:2000, Agrisera, Vännäs, Sweden); anti-UDP glucose pyrophosphorylase (aUGP; AS05 086,1:1500, Agrisera, Vännäs, Sweden); anti-rabbit horse radish peroxidase-conjugated secondary antibody1:100,000; A9169; Sigma, Taufkirchen, Germany); anti-Glutathione S-transferase (aGST, 27-4577-01; 1:2000, Cytiva, Marlborough, MA); anti-rabbit alkaline phosphatase-conjugated secondary antibodies (1:1000, A3937; Sigma, Taufkirchen, Germany)

Validation

anti-S310p/S311p (this study) validated as shown in Figure 5 and Extended Data FIgure 7; for other antibodies see manufacturers' websites

Eukaryotic cell lines

Policy information about cell lines and Sex and Gender in Research

Cell line source(s)

Does not apply

Authentication

Does not apply

Mycoplasma contamination

Does not apply

Commonly misidentified lines
(See ICLAC register)

Does not apply

Palaeontology and Archaeology

Specimen provenance

Does not apply

Specimen deposition

Does not apply

Dating methods

Does not apply

☐ Tick this box to confirm that the raw and calibrated dates are available in the paper or in Supplementary Information.

Ethics oversight

Does not apply

Note that full information on the approval of the study protocol must also be provided in the manuscript.

Animals and other research organisms

Policy information about studies involving animals; ARRIVE guidelines recommended for reporting animal research, and Sex and Gender in Research

Laboratory animals

Does not apply

Wild animals

Does not apply

Reporting on sex

Does not apply

Field-collected samples

Does not apply

Ethics oversight

Does not apply

Note that full information on the approval of the study protocol must also be provided in the manuscript.

Clinical data

Policy information about clinical studies

All manuscripts should comply with the ICMJE guidelines for publication of clinical research and a completed CONSORT checklist must be included with all submissions.

Clinical trial registration	Does not apply
Study protocol	Does not apply
Data collection	Does not apply
Outcomes	Does not apply

Dual use research of concern

Policy information about dual use research of concern

Hazards

Could the accidental, deliberate or reckless misuse of agents or technologies generated in the work, or the application of information presented in the manuscript, pose a threat to:

No	Yes	
☒	☐	Public health
☒	☐	National security
☒	☐	Crops and/or livestock
☒	☐	Ecosystems
☒	☐	Any other significant area

Experiments of concern

Does the work involve any of these experiments of concern:

No	Yes	
☒	☐	Demonstrate how to render a vaccine ineffective
☒	☐	Confer resistance to therapeutically useful antibiotics or antiviral agents
☒	☐	Enhance the virulence of a pathogen or render a nonpathogen virulent
☒	☐	Increase transmissibility of a pathogen
☒	☐	Alter the host range of a pathogen
☒	☐	Enable evasion of diagnostic/detection modalities
☒	☐	Enable the weaponization of a biological agent or toxin
☒	☐	Any other potentially harmful combination of experiments and agents

Plants

Seed stocks	Not of concern
Novel plant genotypes	Not of concern
Authentication	Authenticitation through cell biological analysis, genotyping and phenotyping

ChIP-seq

Data deposition

- [ ] Confirm that both raw and final processed data have been deposited in a public database such as GEO.
- [ ] Confirm that you have deposited or provided access to graph files (e.g. BED files) for the called peaks.

Data access links<br>*May remain private before publication.*	Does not apply
Files in database submission	Does not apply
Genome browser session<br>(e.g. UCSC)	Does not apply

Methodology

Replicates	Does not apply
Sequencing depth	Does not apply
Antibodies	Does not apply
Peak calling parameters	Does not apply
Data quality	Does not apply
Software	Does not apply

Flow Cytometry

Plots

Confirm that:

- [ ] The axis labels state the marker and fluorochrome used (e.g. CD4-FITC).
- [ ] The axis scales are clearly visible. Include numbers along axes only for bottom left plot of group (a 'group' is an analysis of identical markers).
- [ ] All plots are contour plots with outliers or pseudocolor plots.
- [ ] A numerical value for number of cells or percentage (with statistics) is provided.

Methodology

Sample preparation	Does not apply
Instrument	Does not apply
Software	Does not apply
Cell population abundance	Does not apply
Gating strategy	Does not apply

- [ ] Tick this box to confirm that a figure exemplifying the gating strategy is provided in the Supplementary Information.

Magnetic resonance imaging

Experimental design

Design type	Does not apply
Design specifications	Does not apply
Behavioral performance measures	Does not apply

Acquisition

Imaging type(s): Does not apply

Field strength: Does not apply

Sequence & imaging parameters: Does not apply

Area of acquisition: Does not apply

Diffusion MRI ☐ Used ☐ Not used

Preprocessing

Preprocessing software: Does not apply

Normalization: Does not apply

Normalization template: Does not apply

Noise and artifact removal: Does not apply

Volume censoring: Does not apply

Statistical modeling & inference

Model type and settings: Does not apply

Effect(s) tested: Does not apply

Specify type of analysis: ☐ Whole brain ☐ ROI-based ☐ Both

Statistic type for inference (See Eklund et al. 2016): Does not apply

Correction: Does not apply

Models & analysis

n/a	Involved in the study
☐	☐ Functional and/or effective connectivity
☐	☐ Graph analysis
☐	☐ Multivariate modeling or predictive analysis

Functional and/or effective connectivity: Does not apply

Graph analysis: Does not apply

Multivariate modeling and predictive analysis: Does not apply
