# Supplementary figures and images for: D6PK plasma membrane polarity requires a repeated CXX(X)P motif and PDK1-dependent phosphorylation
Source: Nat Plants. 2024 Jan 26;10(2):300–14. doi: 10.1038/s41477-023-01615-6 (PMC10881395; doi:10.1038/s41477-023-01615-6)

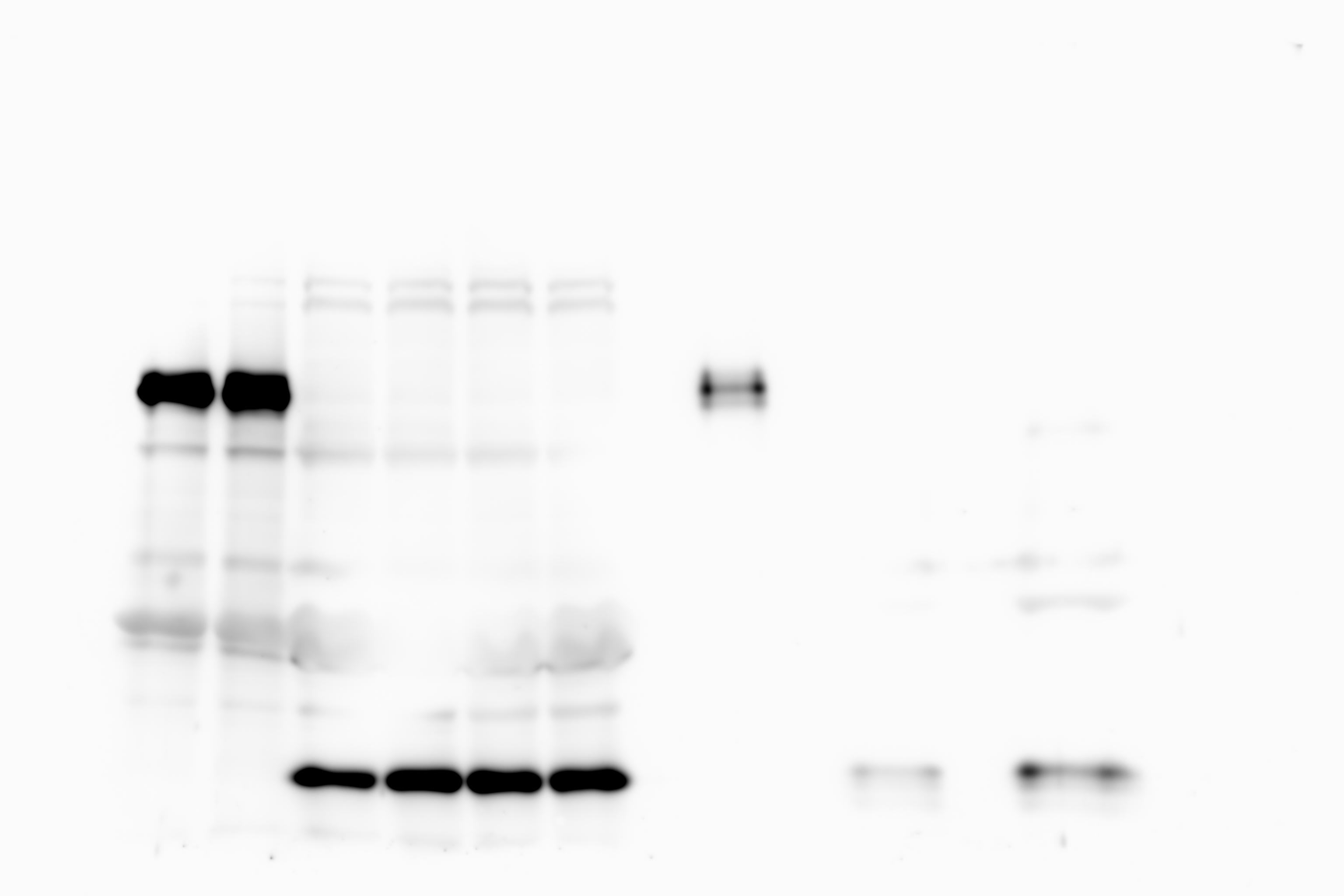

Supplement: Supplementary file 3 — Unprocessed western blots. [file 41477_2023_1615_MOESM3_ESM.tif]

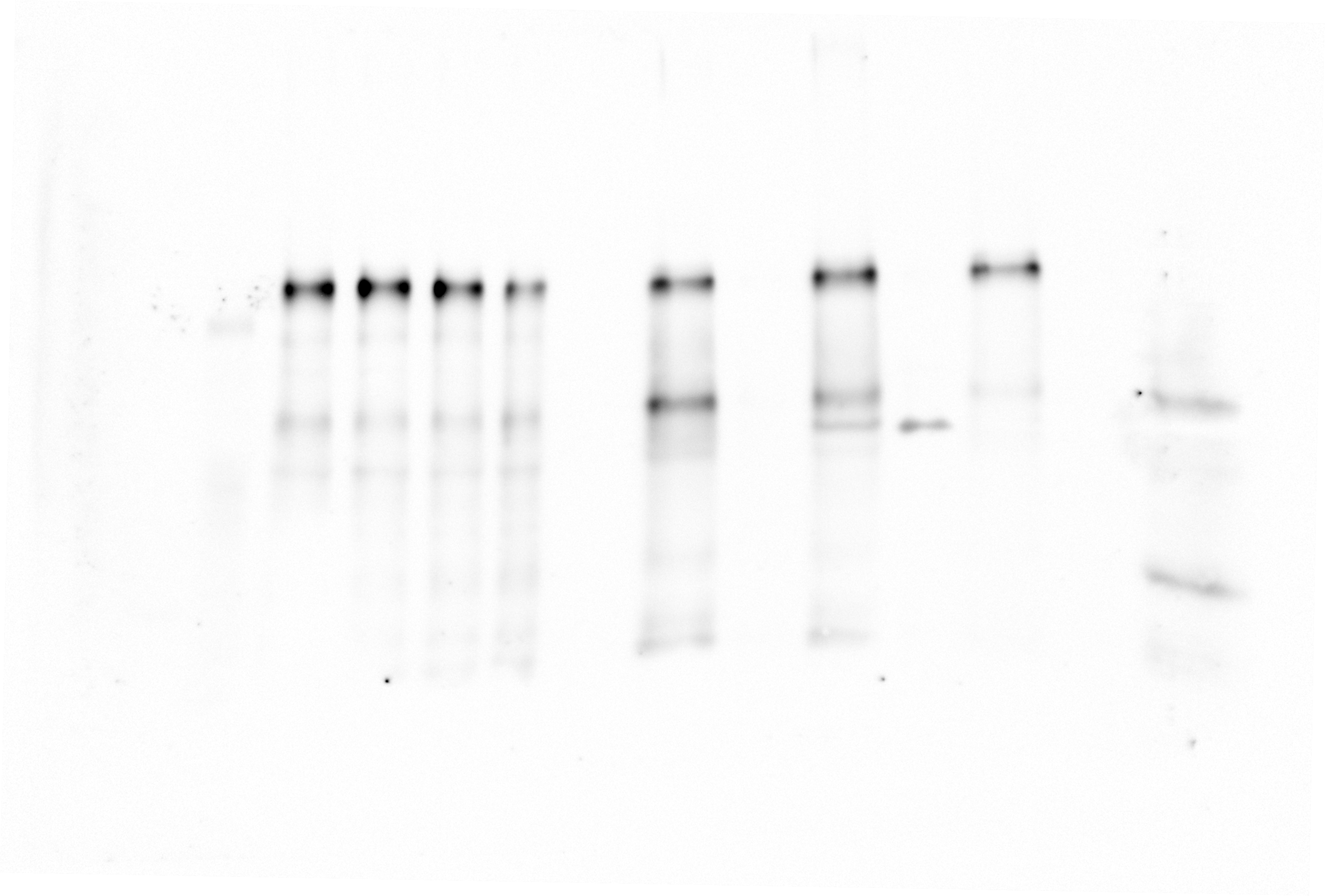

Supplement: Supplementary file 4 — Unprocessed western blots. [file 41477_2023_1615_MOESM4_ESM.tif]

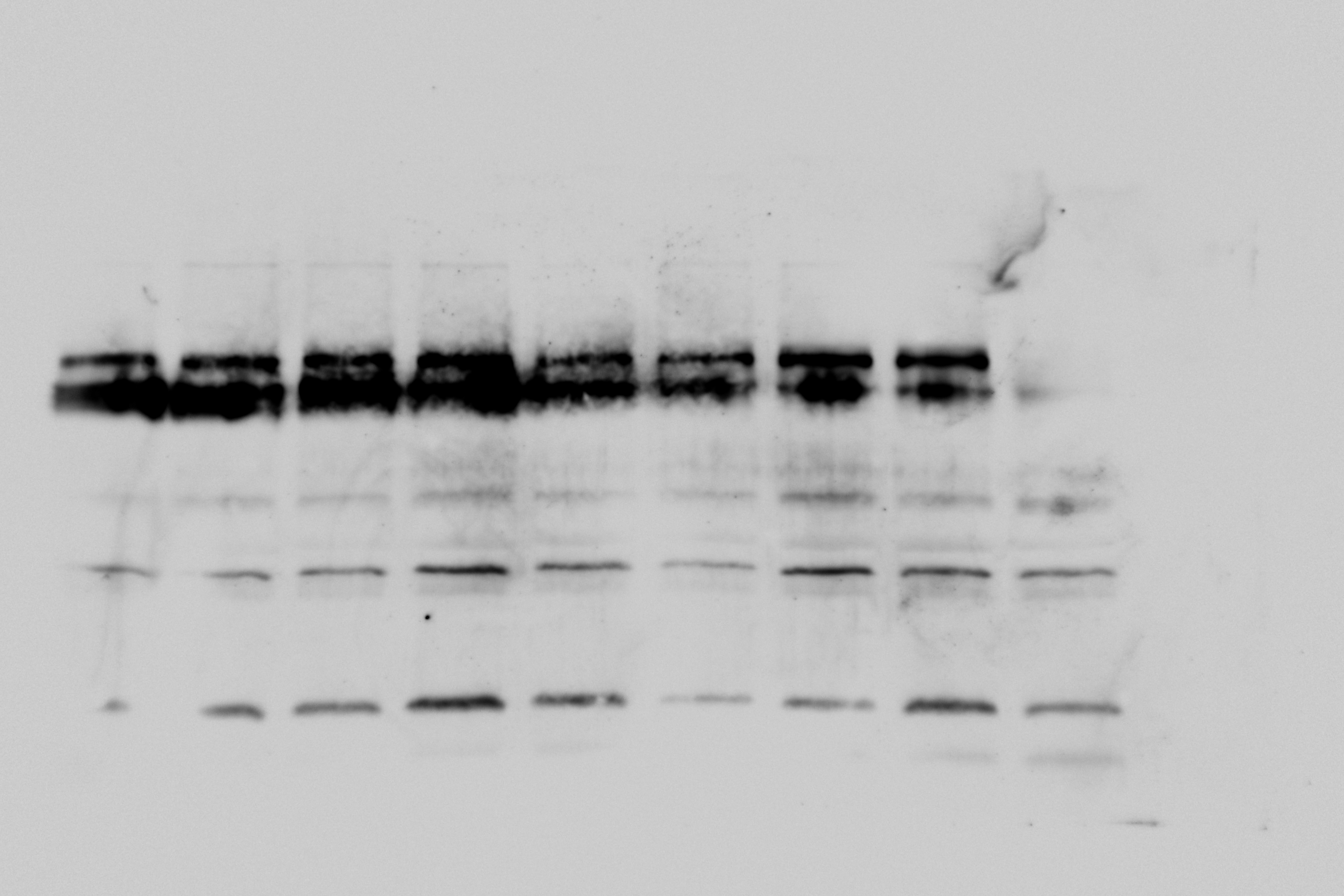

Supplement: Supplementary file 5 — Unprocessed western blots. [file 41477_2023_1615_MOESM5_ESM.tif]
